# Supplementary material for: Smart epidemic control: A hybrid model blending ODEs and agent-based simulations for optimal, real-world intervention planning
Source: PLoS Comput Biol. 2025 May 8;21(5):e1013028. doi: 10.1371/journal.pcbi.1013028 (PMC12061170; doi:10.1371/journal.pcbi.1013028)
Supplement: S1 Text — (PDF) [file pcbi.1013028.s001.pdf]

Supplementary material for

"Smart epidemic control: A hybrid model blending ODEs and agent-based simulations for optimal, real-world intervention planning"

by P. Polcz, I.Z. Reguly, K. Tornai, J. Juhász, S. Pongor, A. Csikász-Nagy, G. Szederkényi

1 Hardware requirements and execution information

PanSim was designed to support large numbers of simulations executing both on personal computers (PC) or computer clusters. In our present work, all simulations were performed on a desktop PC with Intel Core i5-4590 CPU at 3.30 GHz with 32 GBs of DDR3 RAM at 1333 MHz and a GeForce GTX 750 Ti GPU with 2GBs of GDDR5 integrated memory. On this architecture, simulating the spread in a city like Szeged with 180k agents and 81k locations in a 210 days (30 week) long time frame takes approximately 69 seconds. Therefore, generating 1000 simulations for constructing the lookup table requires about 19 hours. We remark that PanSim does not require a GPU necessarily. If the same simulation is executed on a laptop PC with Intel Core i7-4710MQ CPU at 2.50 GHz with 16 GBs of DDR3 RAM at 1600 MHz (without using a GPU), the execution time is about 500 seconds. Implementation details of PanSim are discussed in our earlier papers [1, 2].

2 ODE-based predictions compared to agent-based simulations

Throughout Figures 4–10, we present a control simulation of Scenario A ("Flatten the curve") cycle by cycle, during which the computed measures are recalculated monthly according to the actual outcome of the simulated process (which may be different from that predicted one month before). In each figure, we illustrate the target curve (red), the predicted curve (for the "current future"), the simulation result and its reconstruction. These figures also allow to compare the ODE-based predictions with the simulation results of PanSim.

3 Uncertainty of the lookup table

Specific mobility restriction measures, allow to assess their impact on the number of contacts and hence on the transmission rate – separately for each measure. However, the combined impact of multiple concurrent measures is not straightforward, as their joint effects are often not straightforward to characterize. For instance, intensive testing and contact tracing may have little to no effect in the absence of even mild quarantine policies. The Oxford Stringency Index [3] was a notable attempt to quantify the cumulative impact of various measures, but it relies on predefined heuristics rather than direct estimation from data.

Our approach explicitly estimates the effect of each combination of interventions:

$$I = (\mathrm{TP}, \mathrm{PL}, \mathrm{CF}, \mathrm{SO}, \mathrm{QU}, \mathrm{MA}) \quad \in \quad \mathcal{I} = \{\mathrm{low}, \mathrm{high}\}^3 \times \{\mathrm{low}, \mathrm{medium}, \mathrm{high}\}^3 \tag{1}$$

in terms of a probabilistic transmission rate $\beta(I) \in (0, 1]$, which is estimated by a Gaussian distribution $N(\bar{\beta}(I), \sigma_\beta^2(I))$. Considering the 1000 simulation records (about $237k$ number of simulated days recorder), we computed the mean and standard deviation (Std) of the resulting

transmission rates separately for each of the 216 combination of measures. These values are provided in a supplementary spreadsheet file (Supplement S1 Table).

When our goal is to reduce the maximum possible transmission rate (in a free population, β_{max}) of infections by 30%, i.e., to achieve a rate

$$\beta_{\text{d}} = 0.7 \cdot \beta_{\text{max}}, \tag{2}$$

we obtain a set of feasible combination of measures:

$$\mathcal{I}_{30\%} = \left\{ I \in \mathcal{I} : \beta_{\text{d}} \in \bar{\beta}(I) \pm \sigma_{\beta}(I) \right\}. \tag{3}$$

As the combination of measures achieving a given effect is not necessarily unique, this flexibility allows selecting the measures based on heuristics or specific objectives. For instance, criteria can be introduced to minimize changes in interventions across the intervening cycles. We admit that there are still many opportunities to refine and expand this methodology how to select the "currently best" NPI from $\mathcal{I}_{30\%}$.

Figures 1 and 2 illustrate the obtained mean and Std values for the different NPIS. We note that the "medium" and "low" values for the quarantine policy (QU) resulted in a similar value for mean and Std of the transmission rate. Therefore, solely for visualization purposes, those case with "medium" QU policy are not included in Figures 1 and 2 but used in the control algorithm.

4 Uncertainty analysis of the state reconstruction method

To quantify the accuracy of the state reconstruction, we built a synthetic data set from 1000 simulation records, which were used to construct the lookup table[1] too. This set comprises data frames of about $237k$ simulated days in total. To estimate the uncertainty of the reconstructed value for $\mathbf{L}$ (the number of infected in the latent phase), we approximated separate statistics for multiple admissible intervals for $\mathbf{L}$ (according to the moving window principle). Namely, the reconstruction error when $\mathbf{L} = L_0$ was characterized using the data points falling into the interval $L_0 \pm \Delta L$. We repeated this study for compartments $\mathbf{P}$, $\mathbf{A}$, $\mathbf{I}$, and $\mathbf{S}$. The distribution of the 237k samples and the expected state estimation error for the different ranges of $\mathbf{I}$, $\mathbf{P}$, $\mathbf{A}$, $\mathbf{I}$, and $\mathbf{S}$ are illustrated in Figure 11. Figures 12–25 visualize the reconstruction accuracy for multiple different outbreaks simulated.

References

[1] I. Z. Reguly et al. "Microsimulation based quantitative analysis of COVID-19 management strategies". In: *PLOS Computational Biology* 18.1 (Jan. 2022), pp. 1–14. DOI: 10.1371/journal.pcbi.1009693.

[2] B. Keomley-Horvath et al. "The design and utilisation of PanSim, a portable pandemic simulator". In: *2022 First Combined International Workshop on Interactive Urgent Supercomputing (CIW-IUS)*. IEEE, Nov. 2022. DOI: 10.1109/ciw-ius56691.2022.00006.

[3] T. Hale et al. "A global panel database of pandemic policies (Oxford COVID-19 government response tracker)". In: *Nature Human Behaviour* 5.4 (Mar. 2021), pp. 529–538. DOI: 10.1038/s41562-021-01079-8.

[1] for more details, we refer to Subsection "Construct a lookup table" in Section METHODS of the main text

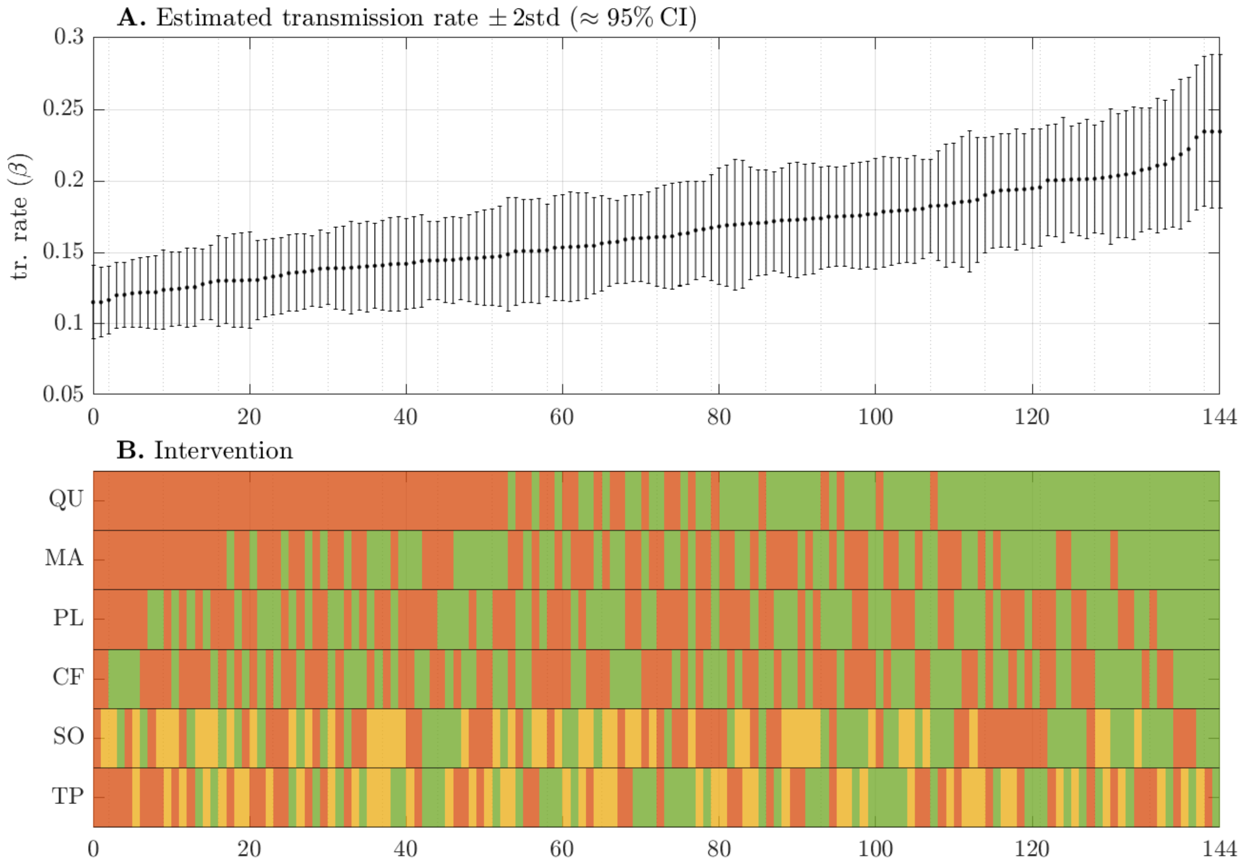

Figure 1: Mean and variance of the estimated transmission rates β for the different combination of measures ordered with respect to the obtained mean value for β.

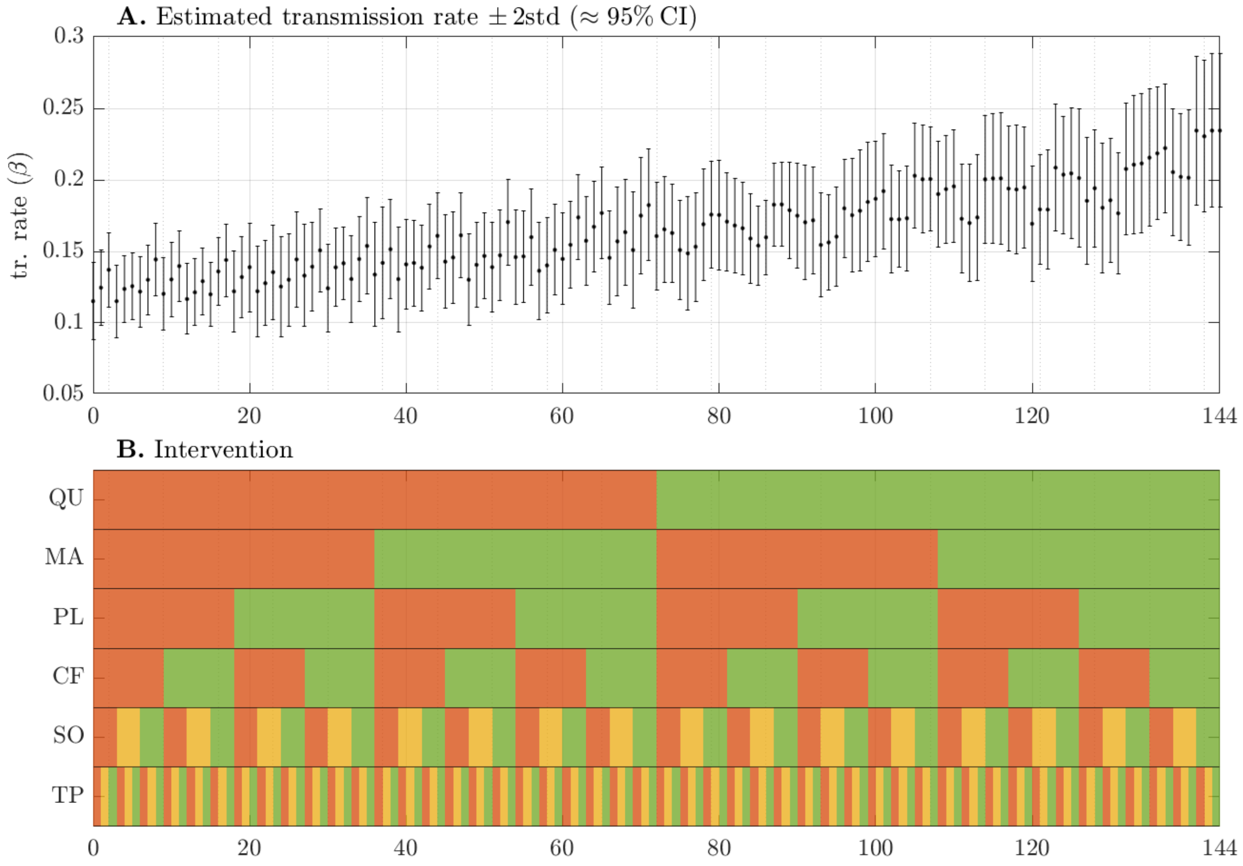

Figure 2: Mean and variance of the estimated transmission rates β for the different combination of measures ordered with respect to the stringency of the measures.

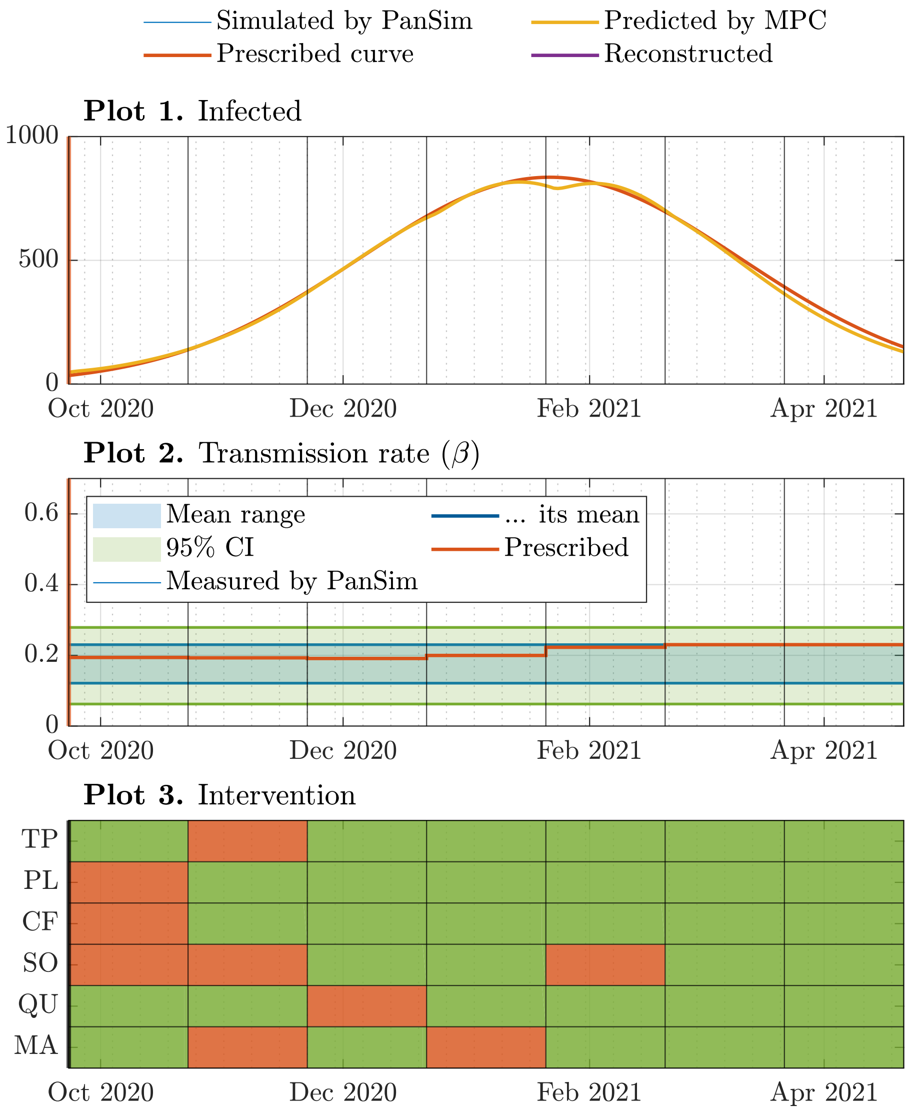

Figure 3: Control simulation before the first intervention planing cycle. The red bell-shaped curve illustrates the prescribed trajectory for the infected people with symptoms in the main phase of the disease ($\mathbf{I}$). The yellow curve, which aims to follow the reference curve is trajectory for $\mathbf{I}$ predicted by the discrete-time ODE (i.e., the recursion) model 2. In Plot 2. the red staircase function illustrates the necessary transmission rate β to achieve the prescribed curve for $\mathbf{I}$. The blue area in Plot 2 highlights the range of the computed mean values for β for the different interventions, whereas, the green area illustrates the range of the mean $\pm$ Std computed for the different interventions. Plot 3 illustrates the stringency (low: green, medium: yellow, high: red) planned for the six independent measures summarized in Table 1.

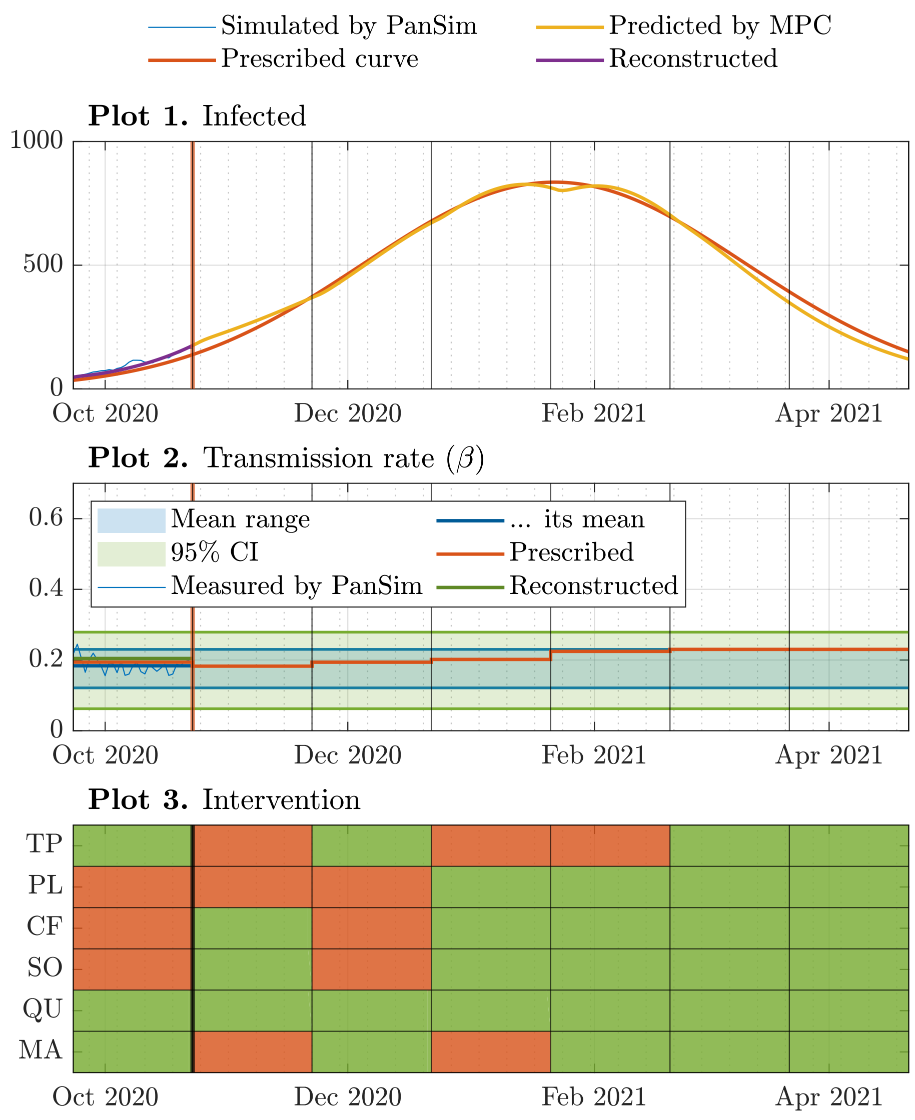

Figure 4: Control simulation before the second intervention planing cycle. The vertical red line illustrates the current time of the simulation, which is now at day $h = 30\,[\text{days}]$. The light blue signals in Plot 1 and Plot 2 illustrate the simulated value of $\mathbf{I}$ and the numerically approximated transmission rate of the simulated spread. Purple signal in Plot 1 constitutes the reconstructed number of infected $\mathbf{I}$ if a constant transmission rate is assumed for the first cycle (solid green segment in Plot 2). The reference trajectory (red bell-shaped curve) is the same as in Figure 4, but the prediction made from day 30 for the remaining $T = 6 \times h = 180\,[\text{days}]$ is slightly different from that in Figure 4. This is due to the fact that in the first month the simulated epidemic process did not follow the predicted curve exactly. Accordingly, the transmission rates and the interventions, respectively, are recalculated from day 31.

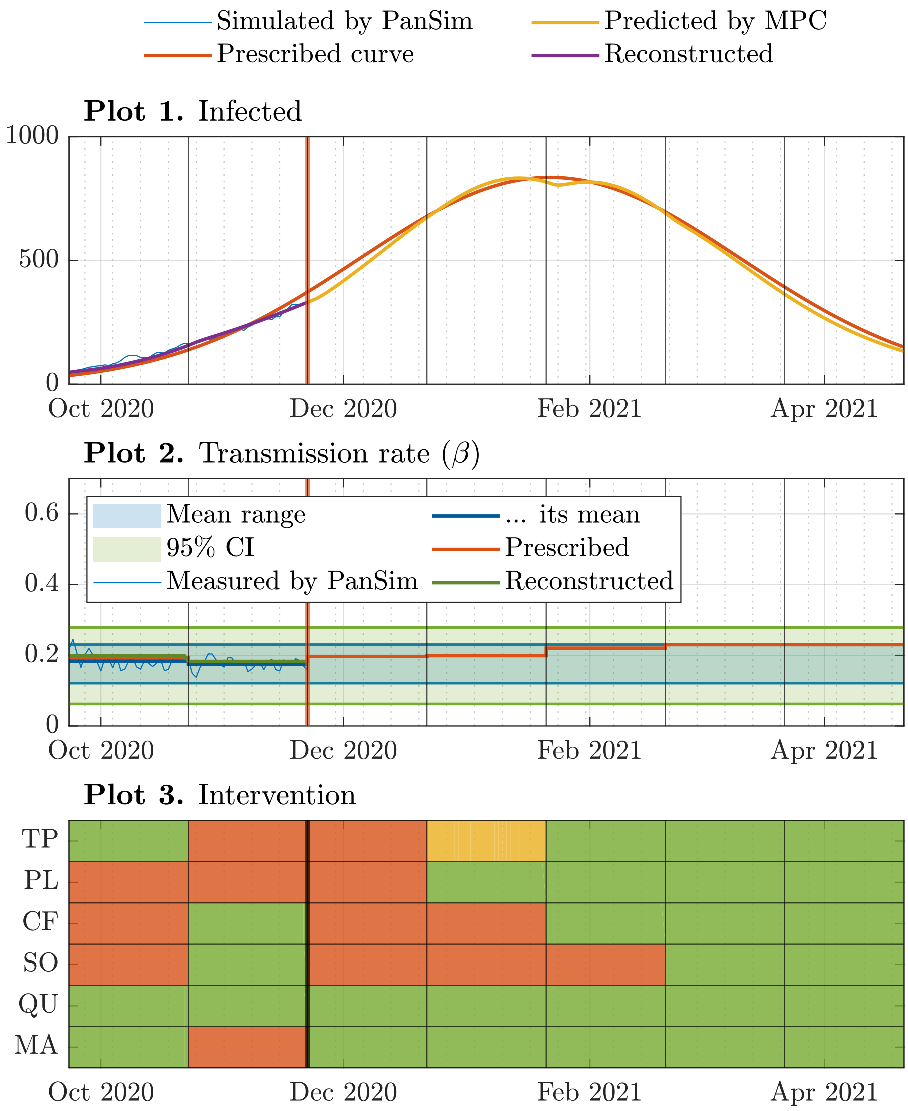

Figure 5: Control simulation before the third intervention planing cycle. The vertical red line illustrates the current time of the simulation, which is now at day 60. The light blue signals in Plot 1 and Plot 2 illustrate the simulated value of $\mathbf{I}$ and the numerically approximated transmission rate of the simulated spread. Purple signal in Plot 1 constitutes the reconstructed number of infected $\mathbf{I}$ if a constant (but potentially different) transmission rate is assumed for the first two cycles (solid green staircase function in Plot 2). The reference trajectory (red bell-shaped curve) is the same as in Figure 4, but the prediction made from day 60 for the remaining $T = 5 \times h = 150\,[\text{days}]$ is slightly different from that in Figure 4. This is due to the fact that in the second cycle the simulated epidemic process did not follow the curve predicted at day 30 exactly. Accordingly, the transmission rates and the interventions, respectively, are recalculated from day 61.

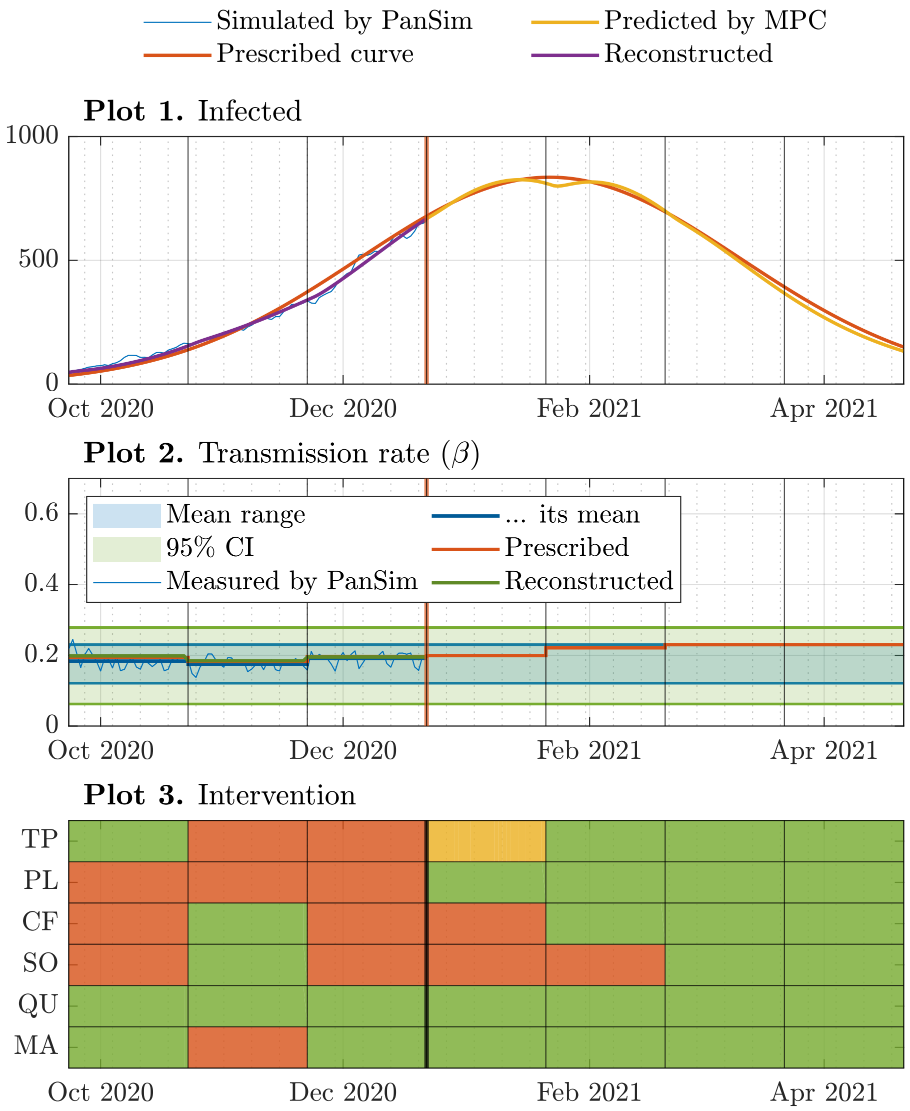

Figure 6: Control simulation before the 4th intervention planing cycle. The vertical red line illustrates the current time of the simulation. We refer to Figures 4, 4, 5 for more details.

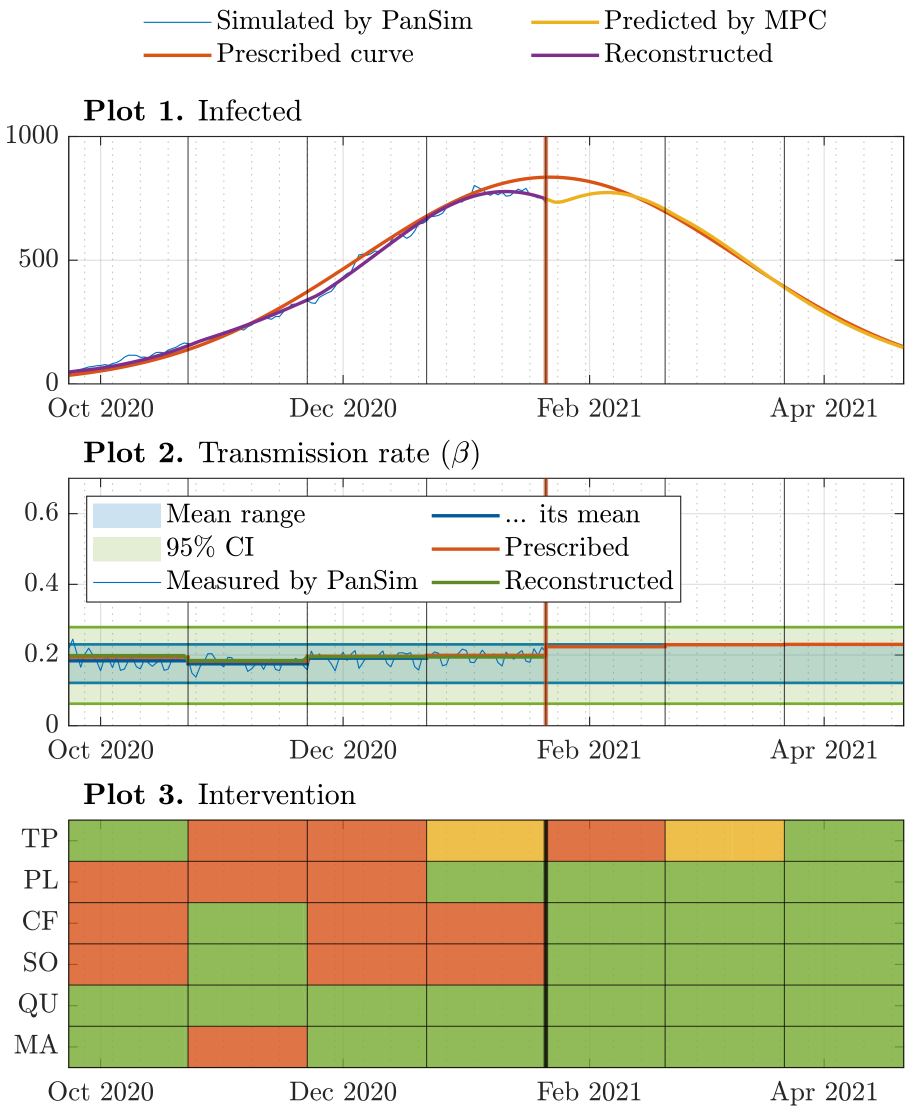

Figure 7: Control simulation before the 5th intervention planing cycle. The vertical red line illustrates the current time of the simulation. We refer to Figures 4–6 for more details.

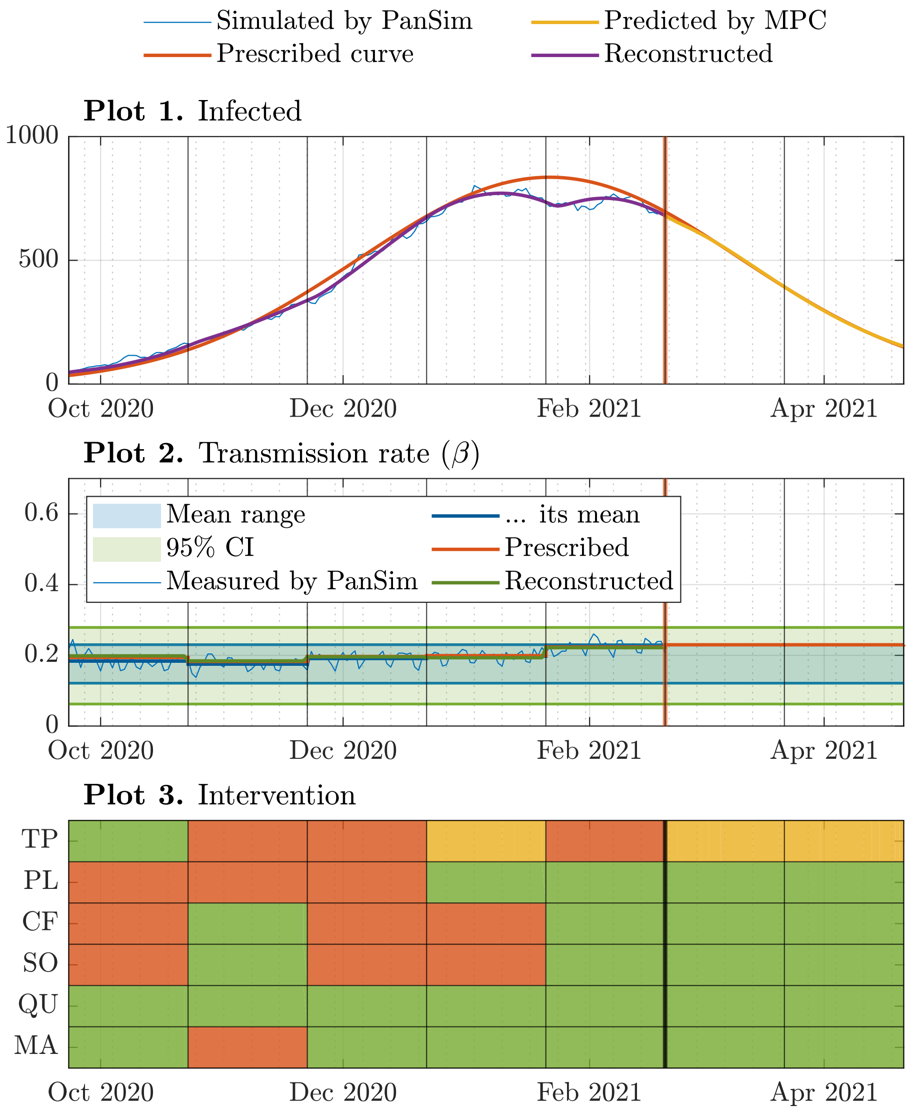

Figure 8: Control simulation before the 6th intervention planing cycle. The vertical red line illustrates the current time of the simulation. We refer to Figures 4–7 for more details.

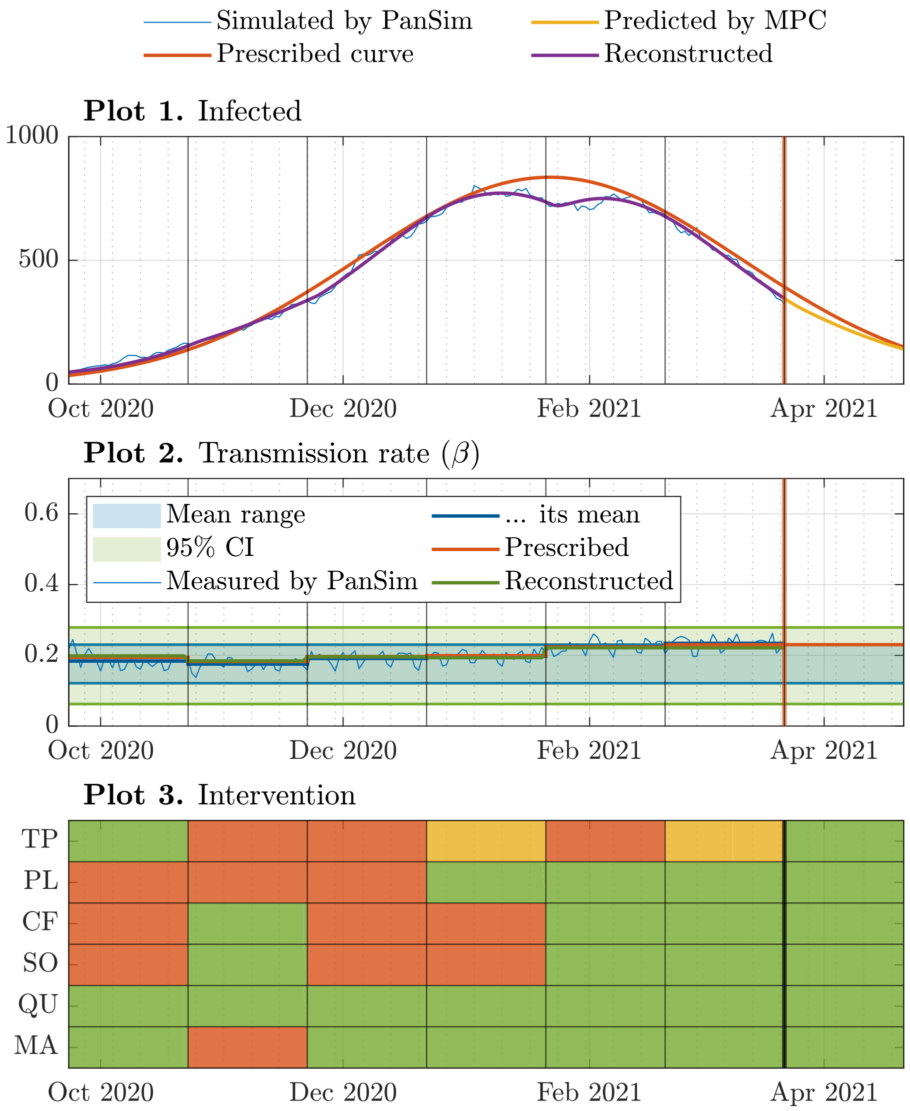

Figure 9: Control simulation before the 7th intervention planing cycle. The vertical red line illustrates the current time of the simulation. We refer to Figures 4–8 for more details.

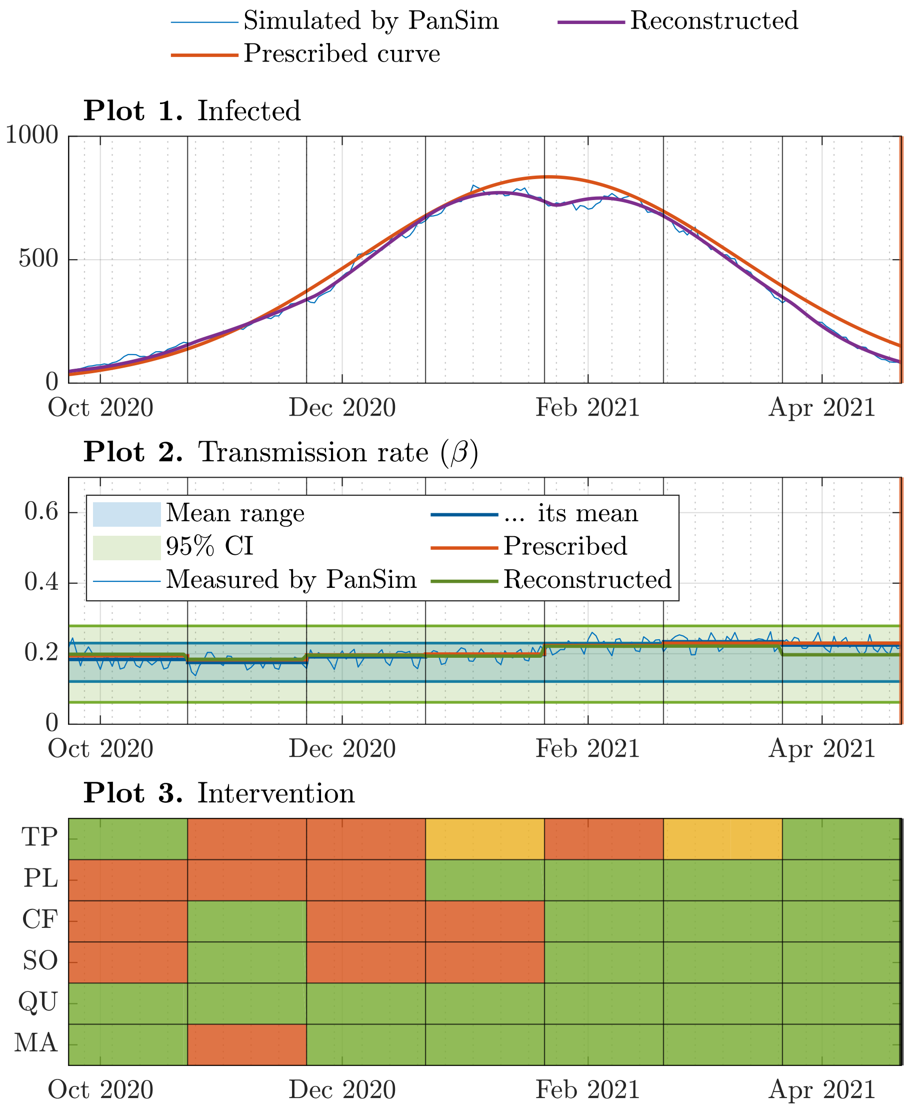

Figure 10: Control simulation after the 7th intervention planing cycle. The vertical red line illustrates the current time of the simulation. We refer to Figures 4–9 for more details.

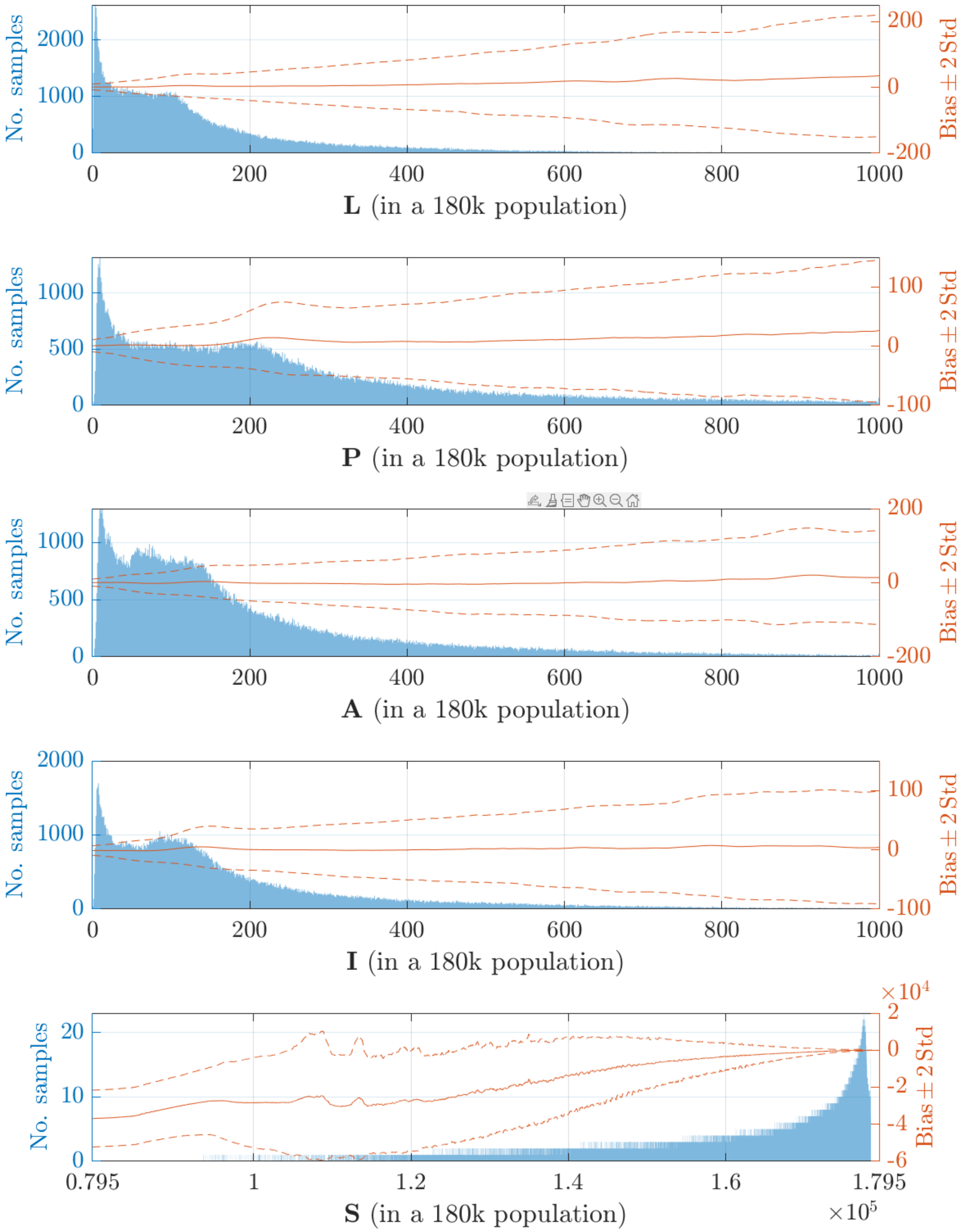

Figure 11: Distribution of samples and the uncertainty of the reconstruction. The histogram shows, how the 236,935 samples are distributed with respect to the distinct values of the simulated quantities **L**, **P**, **A**, **I**, **S** (left axes). The solid lines illustrates the average bias error of the reconstruction, whereas, the pairs of dashed lines bound the 95% CI of the bias error (right axes). E.g., the third plot illustrates that the reconstruction of **A** is about 1% biased and the relative error is less than 12%.

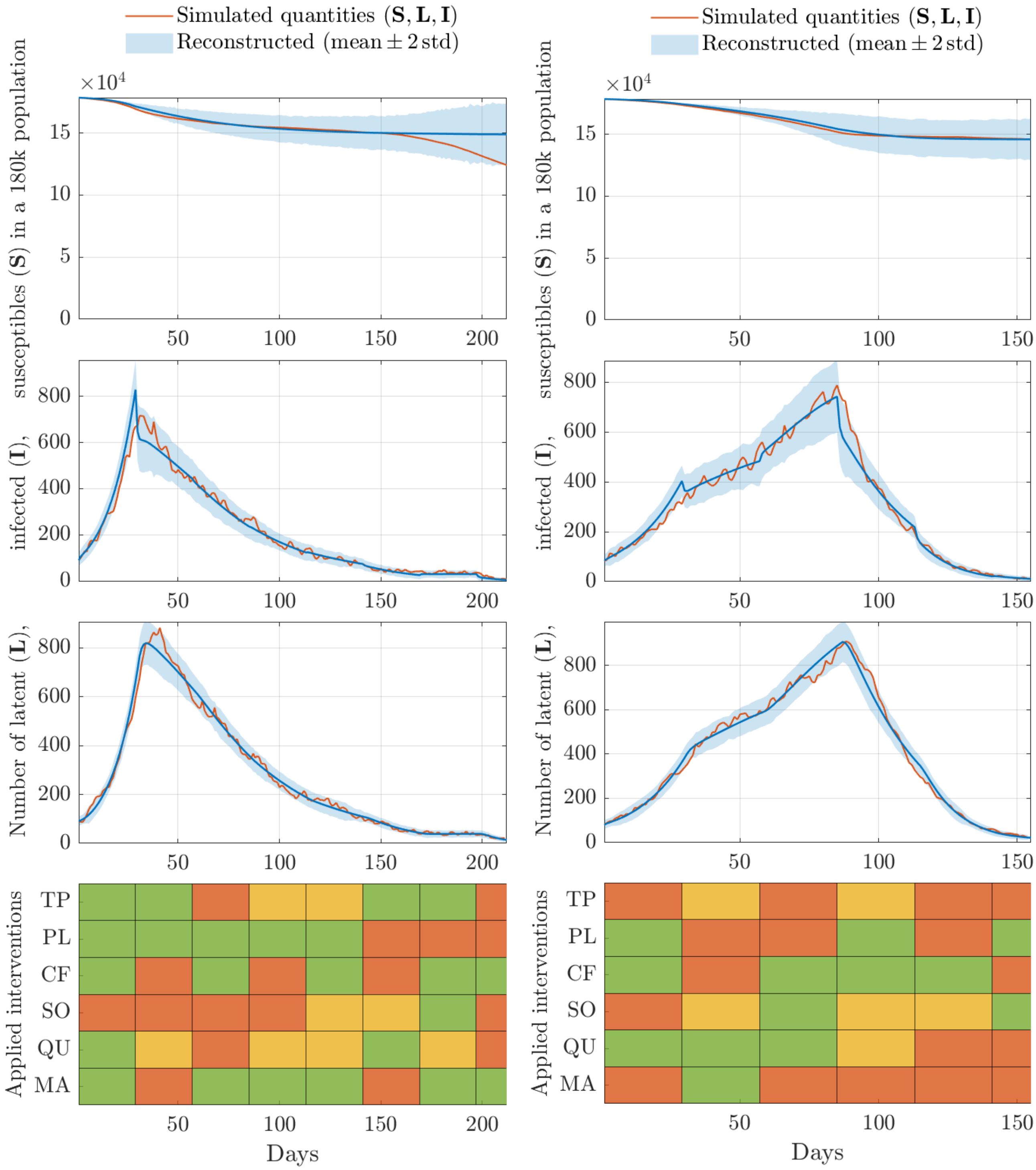

Figure 12: Estimated reconstruction error for two different outbreak scenarios simulated with two different sequence of interventions. The estimated 95% CI of the reconstruction is illustrated by the blue area.

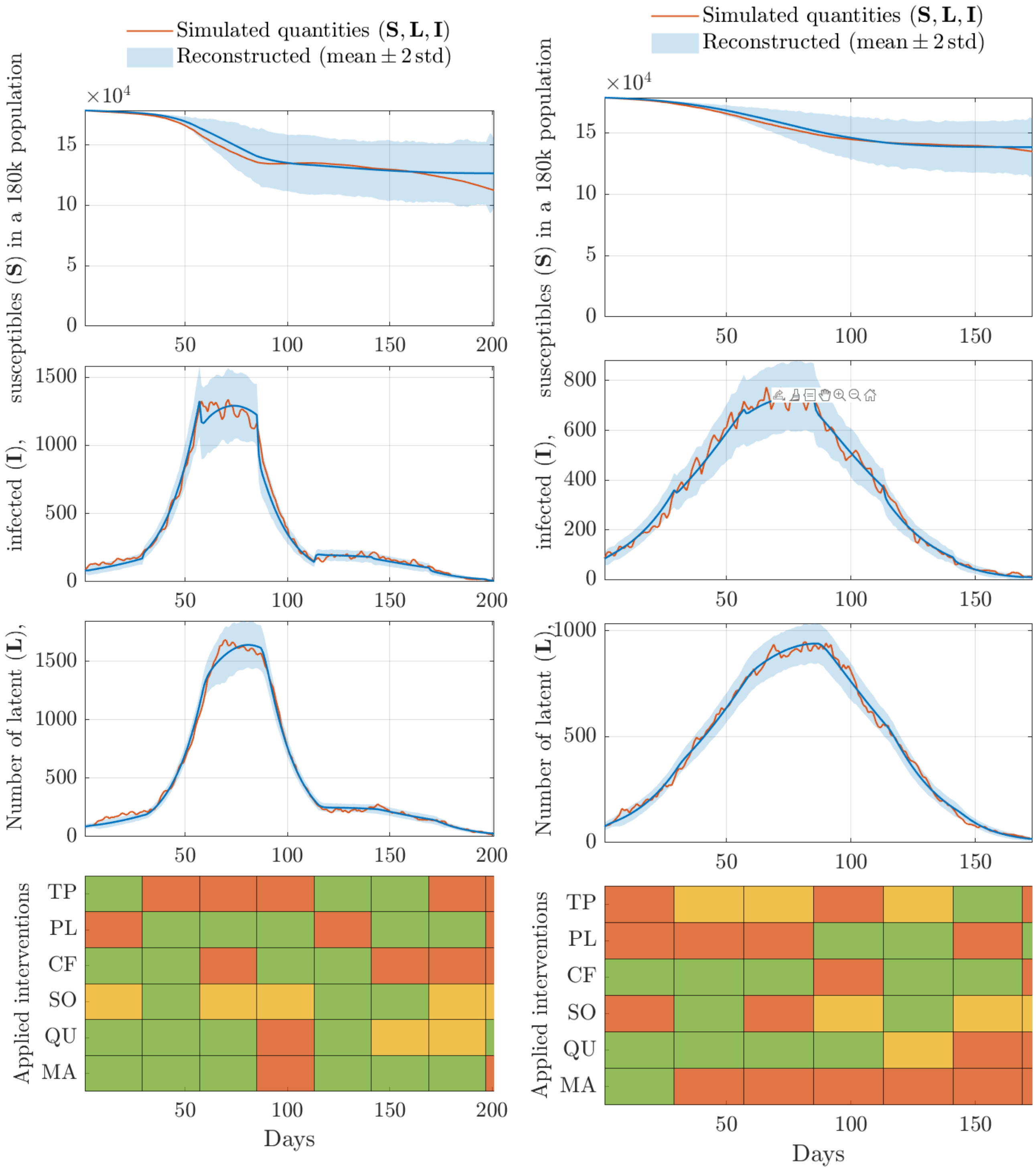

Figure 13: Estimated reconstruction error for two different outbreak scenarios simulated with two different sequence of interventions. The estimated 95% CI of the reconstruction is illustrated by the blue area.

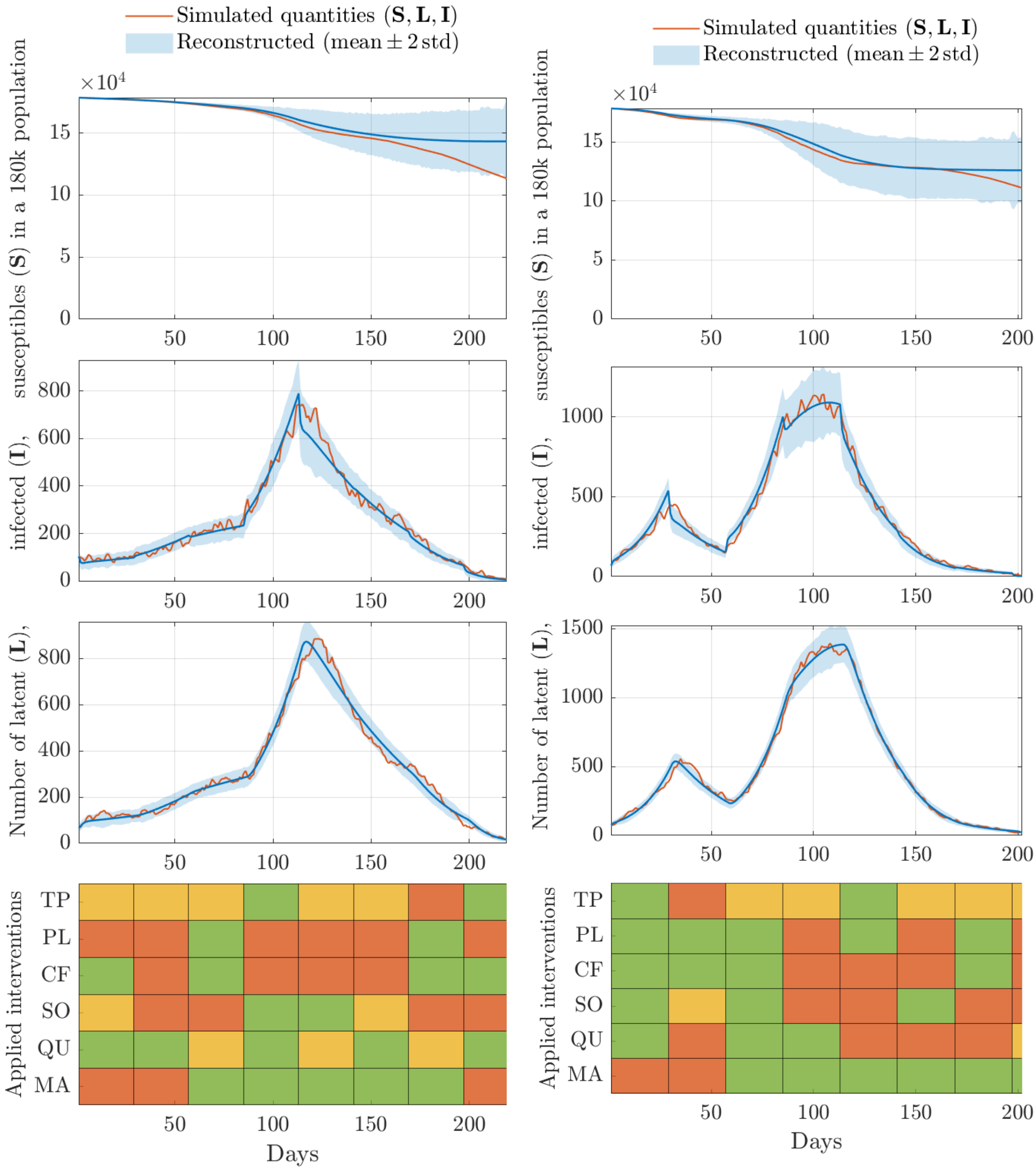

Figure 14: Estimated reconstruction error for two different outbreak scenarios simulated with two different sequence of interventions. The estimated 95% CI of the reconstruction is illustrated by the blue area.

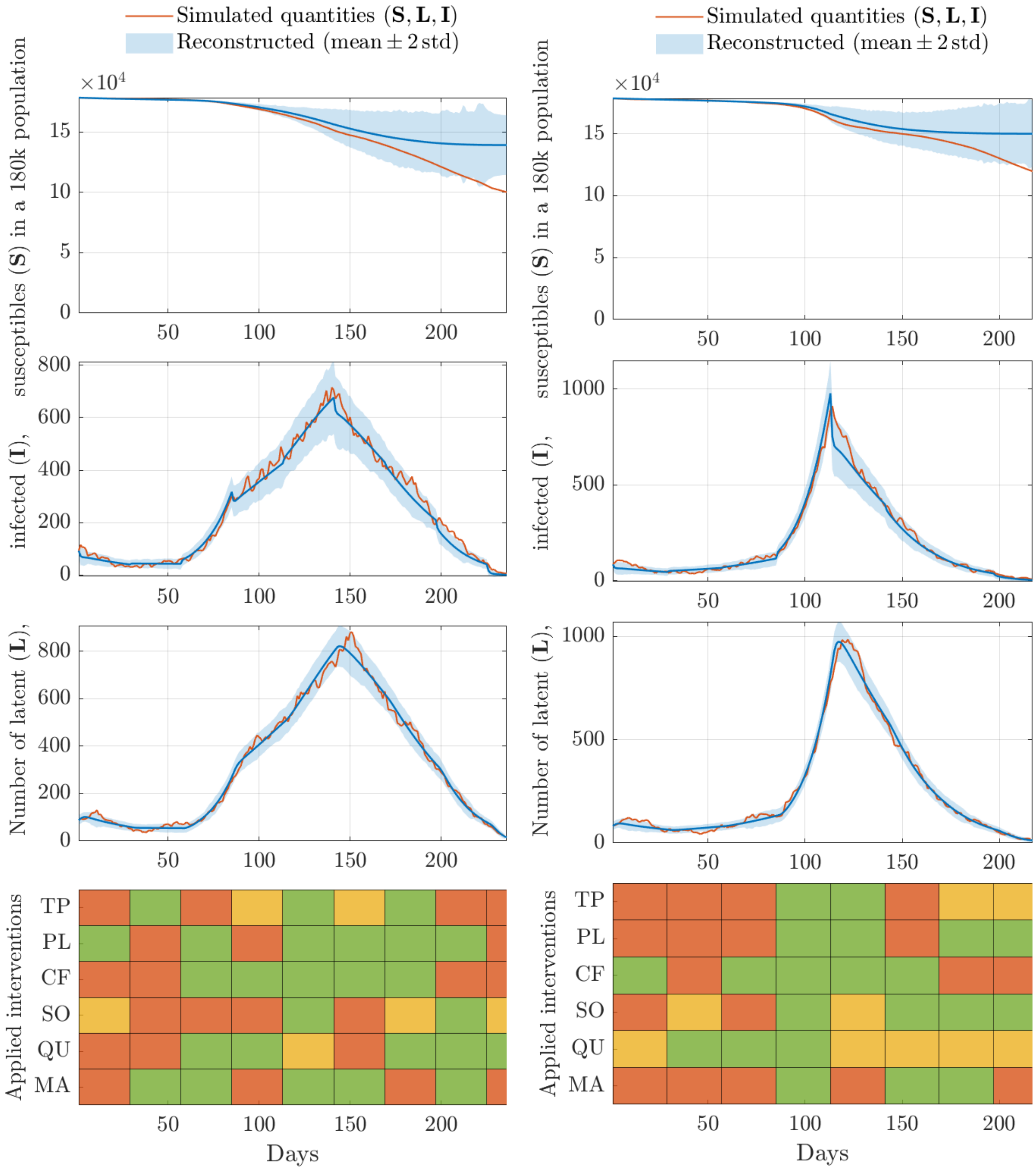

Figure 15: Estimated reconstruction error for two different outbreak scenarios simulated with two different sequence of interventions. The estimated 95% CI of the reconstruction is illustrated by the blue area.

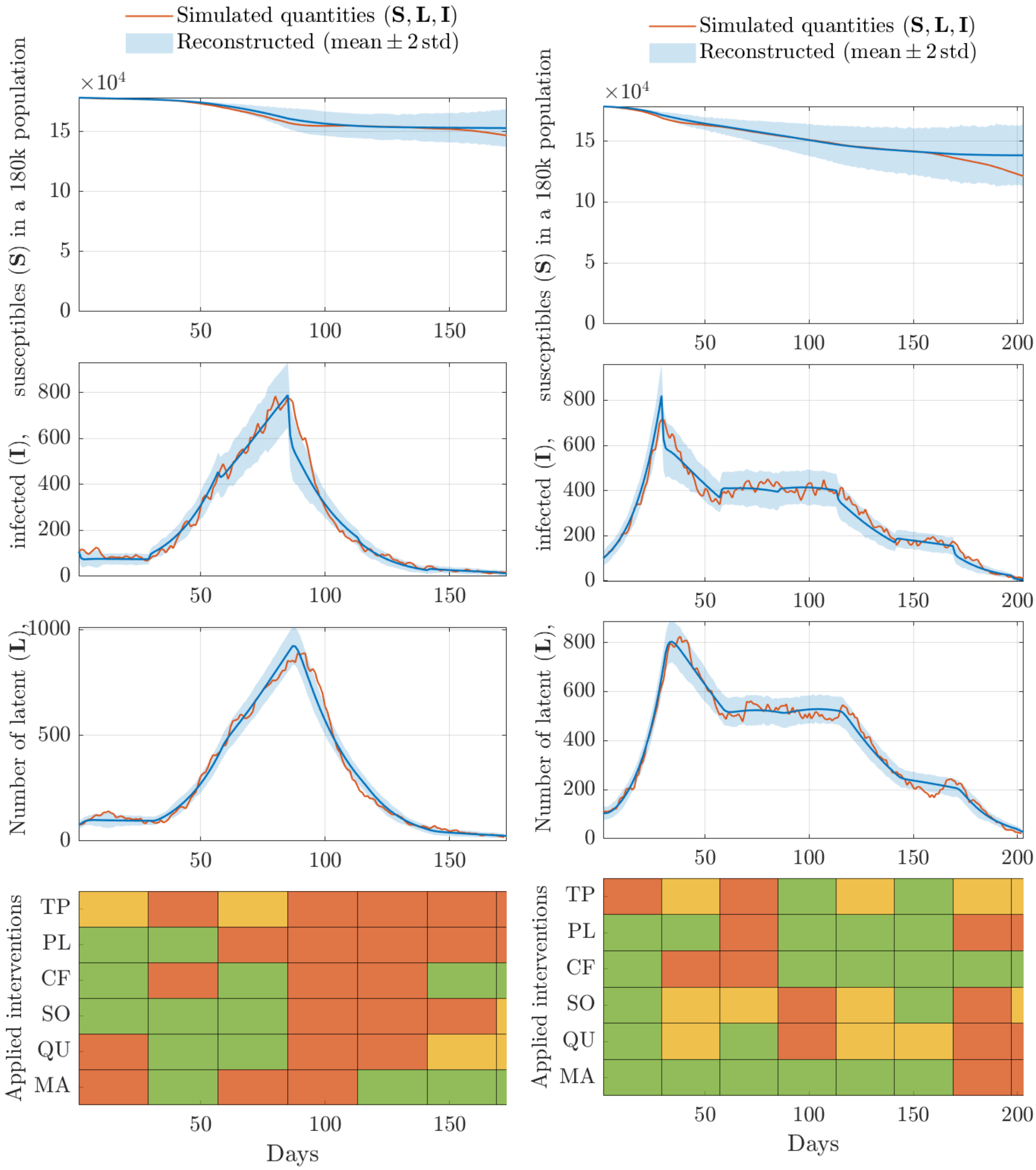

Figure 16: Estimated reconstruction error for two different outbreak scenarios simulated with two different sequence of interventions. The estimated 95% CI of the reconstruction is illustrated by the blue area.

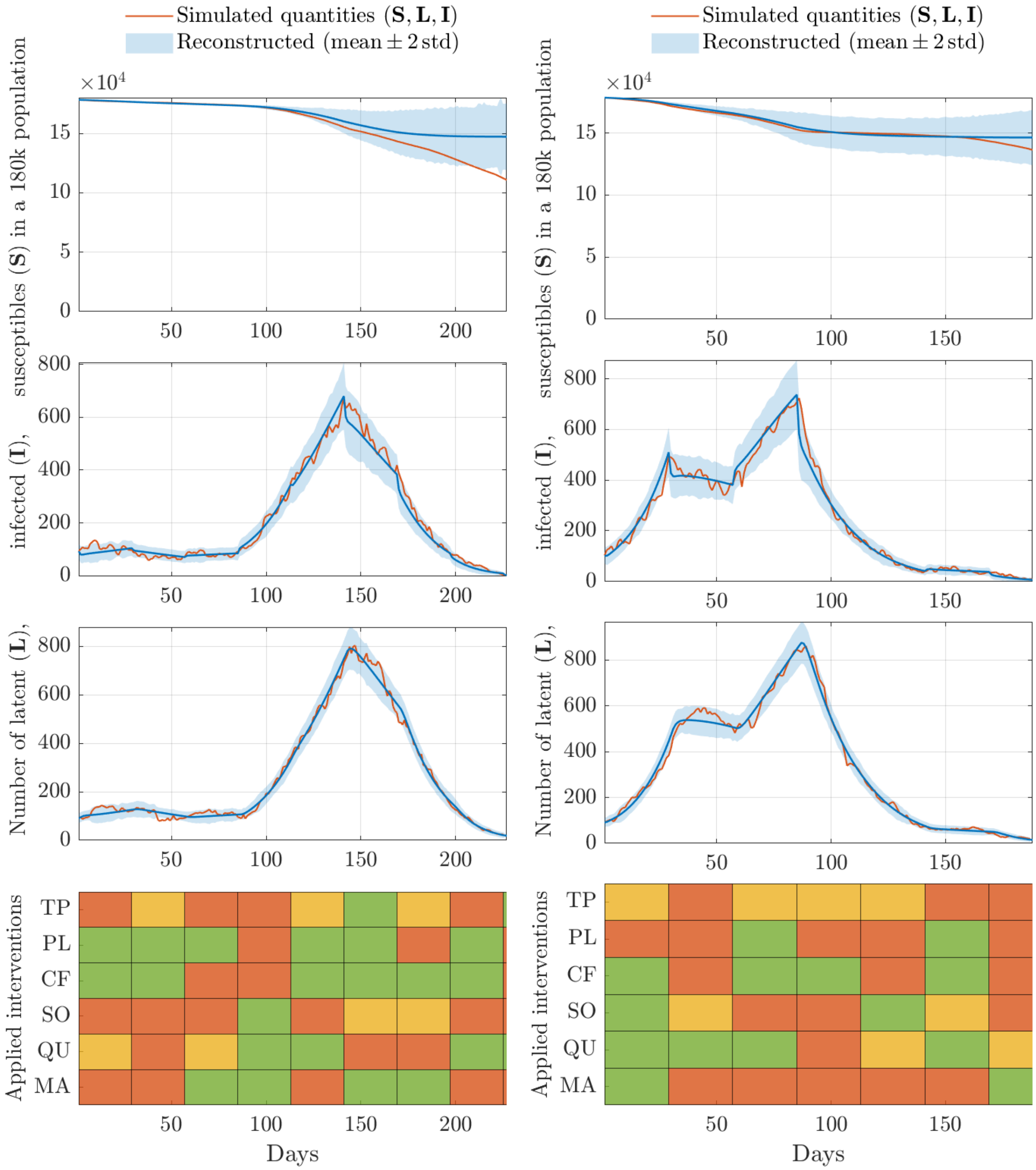

Figure 17: Estimated reconstruction error for two different outbreak scenarios simulated with two different sequence of interventions. The estimated 95% CI of the reconstruction is illustrated by the blue area.

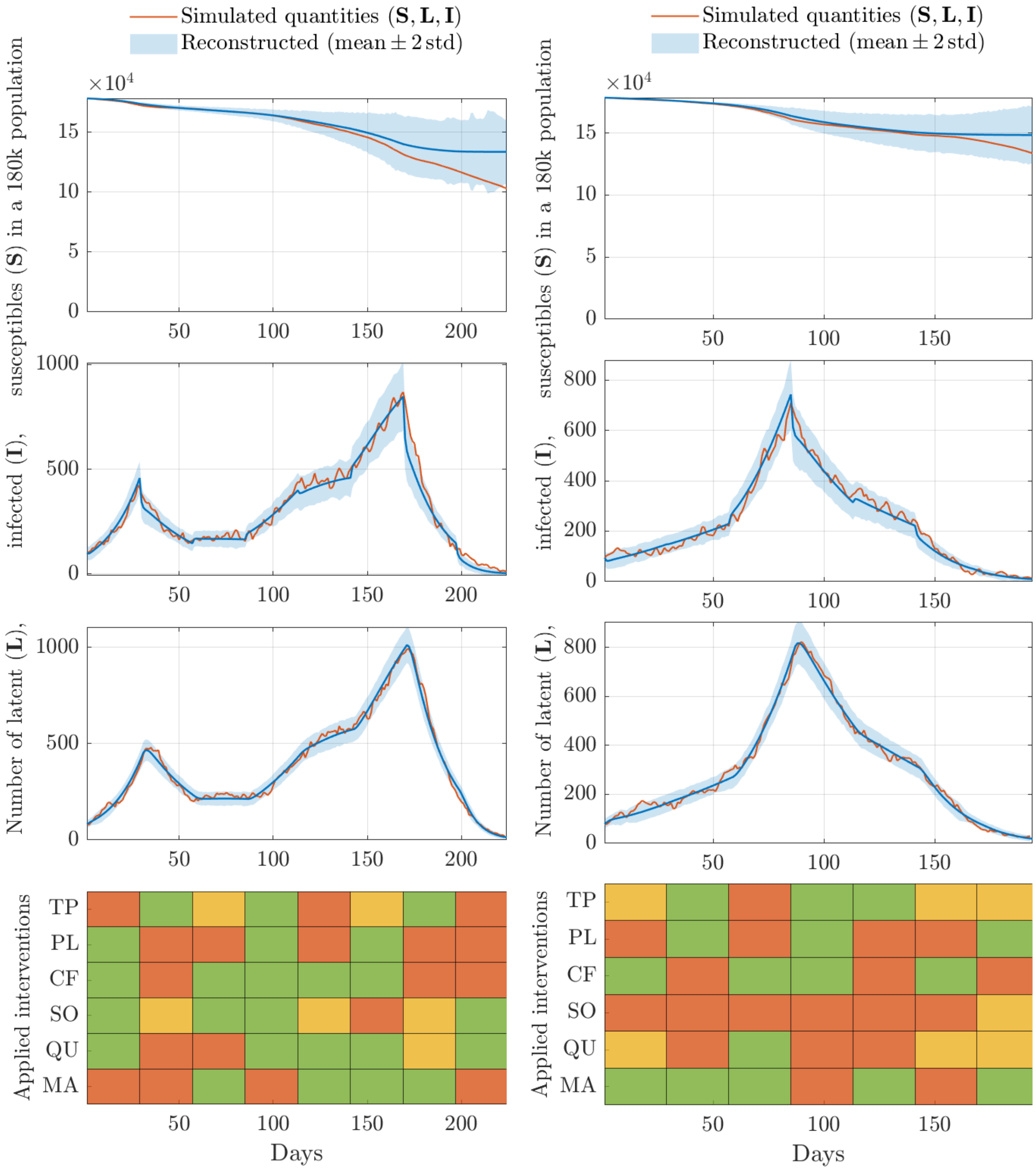

Figure 18: Estimated reconstruction error for two different outbreak scenarios simulated with two different sequence of interventions. The estimated 95% CI of the reconstruction is illustrated by the blue area.

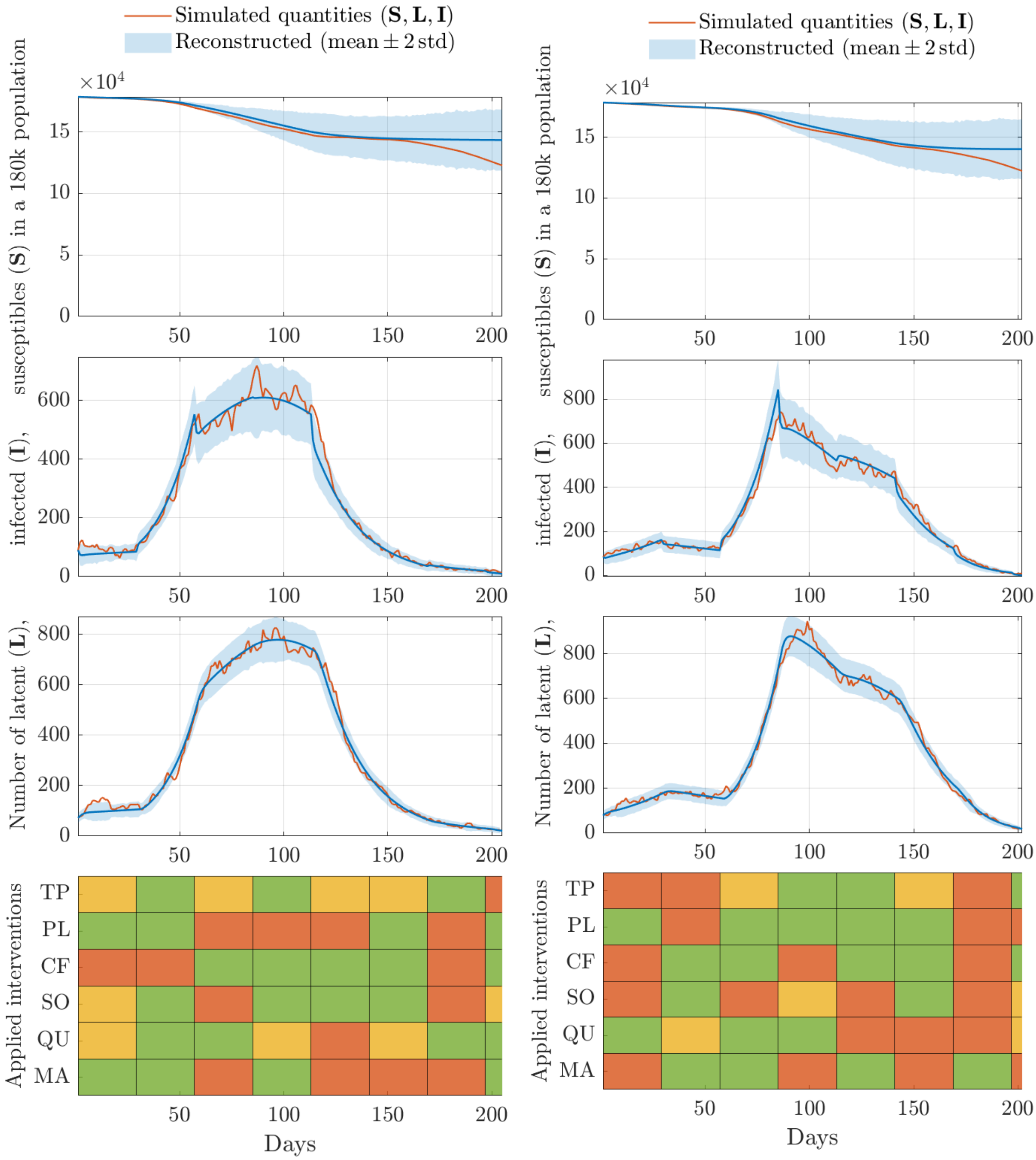

Figure 19: Estimated reconstruction error for two different outbreak scenarios simulated with two different sequence of interventions. The estimated 95% CI of the reconstruction is illustrated by the blue area.

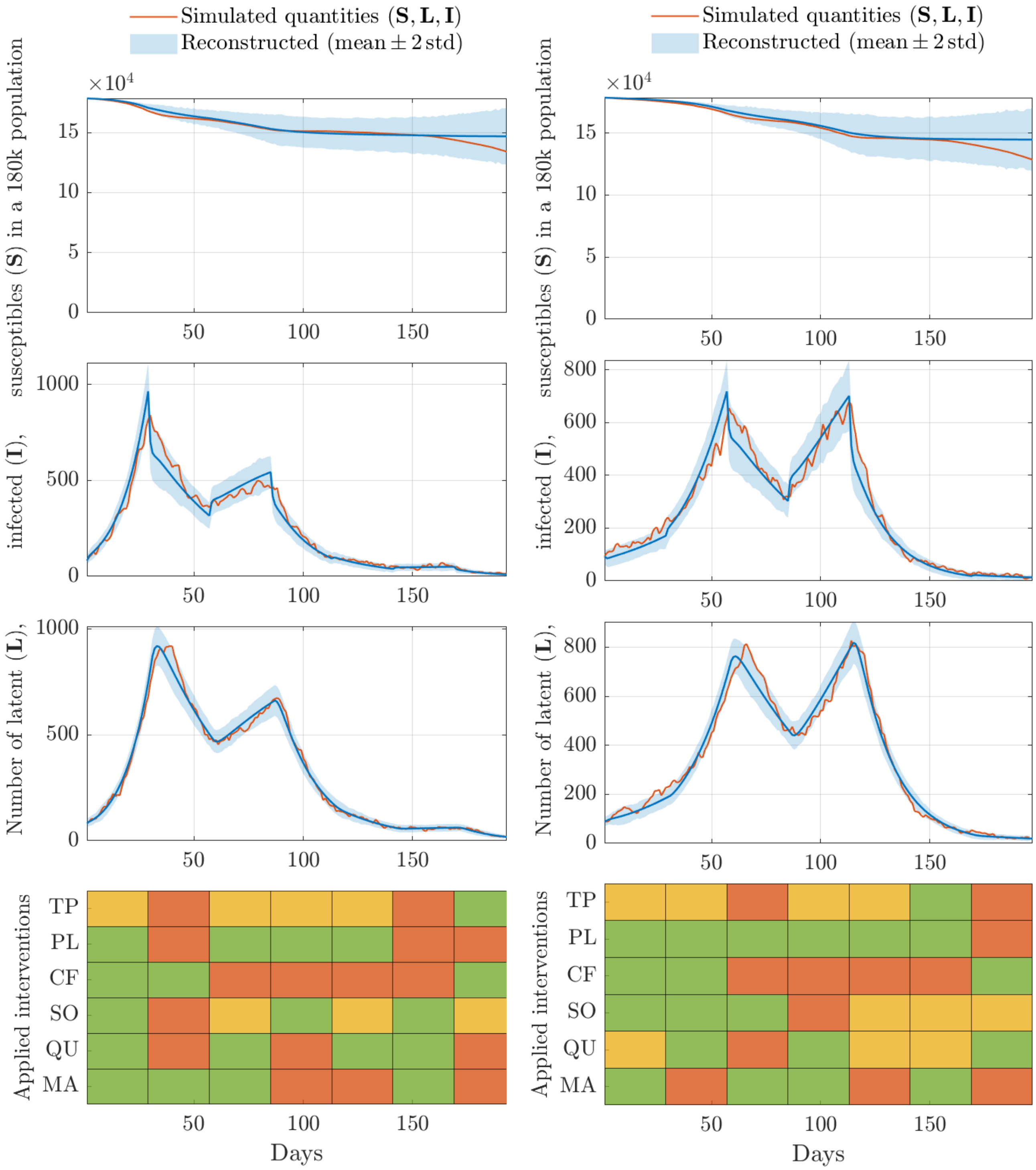

Figure 20: Estimated reconstruction error for two different outbreak scenarios simulated with two different sequence of interventions. The estimated 95% CI of the reconstruction is illustrated by the blue area.

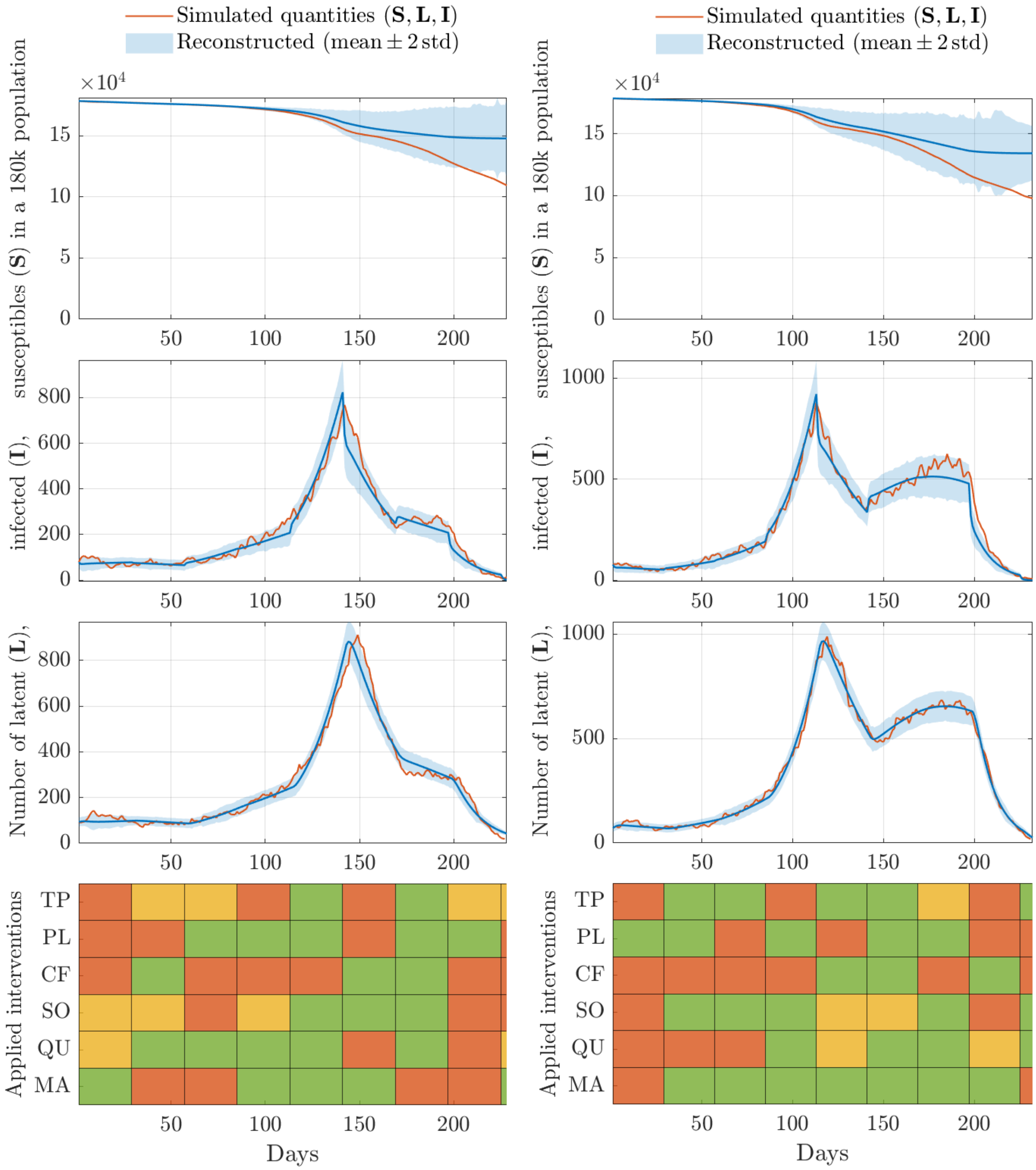

Figure 21: Estimated reconstruction error for two different outbreak scenarios simulated with two different sequence of interventions. The estimated 95% CI of the reconstruction is illustrated by the blue area.

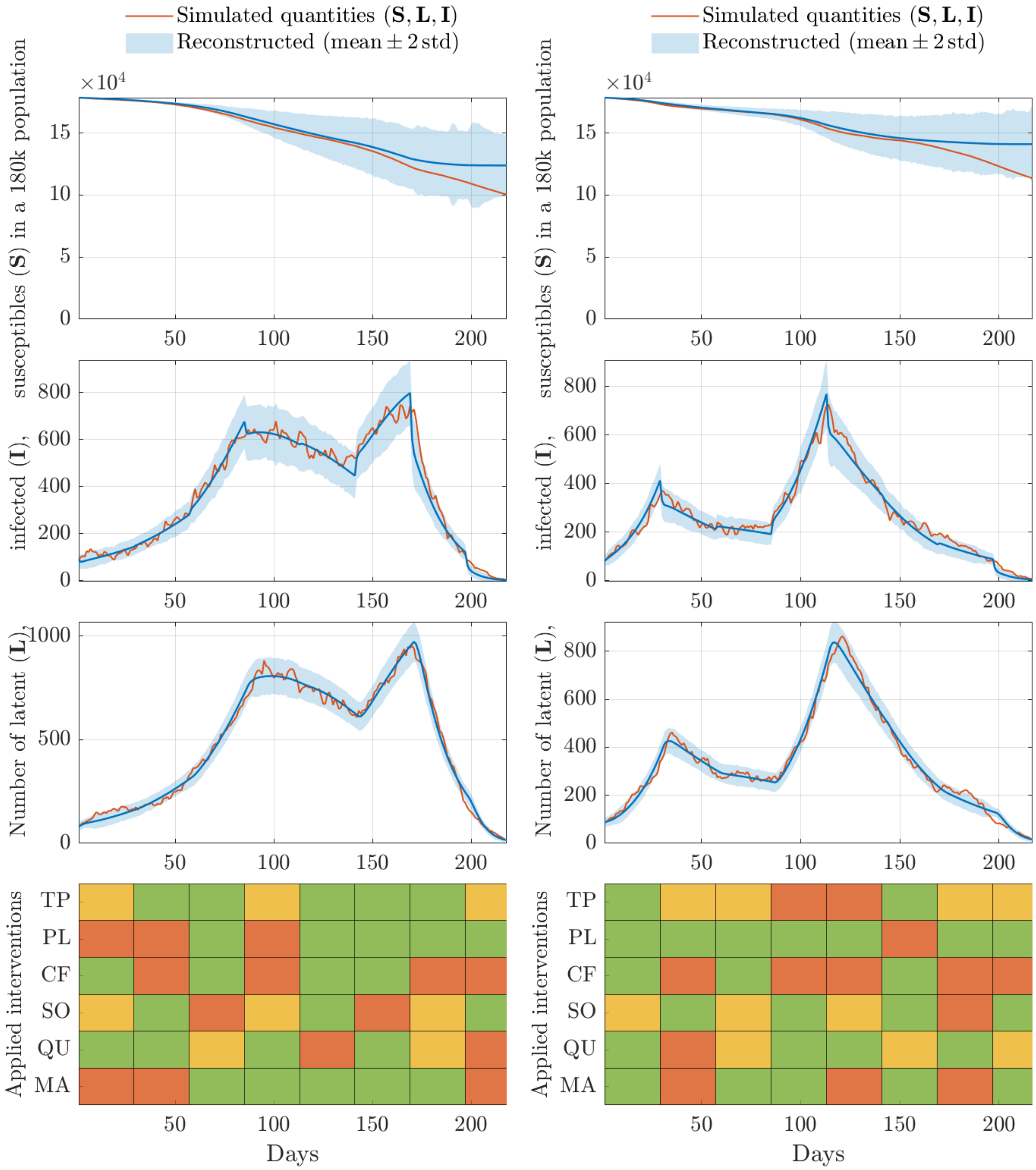

Figure 22: Estimated reconstruction error for two different outbreak scenarios simulated with two different sequence of interventions. The estimated 95% CI of the reconstruction is illustrated by the blue area.

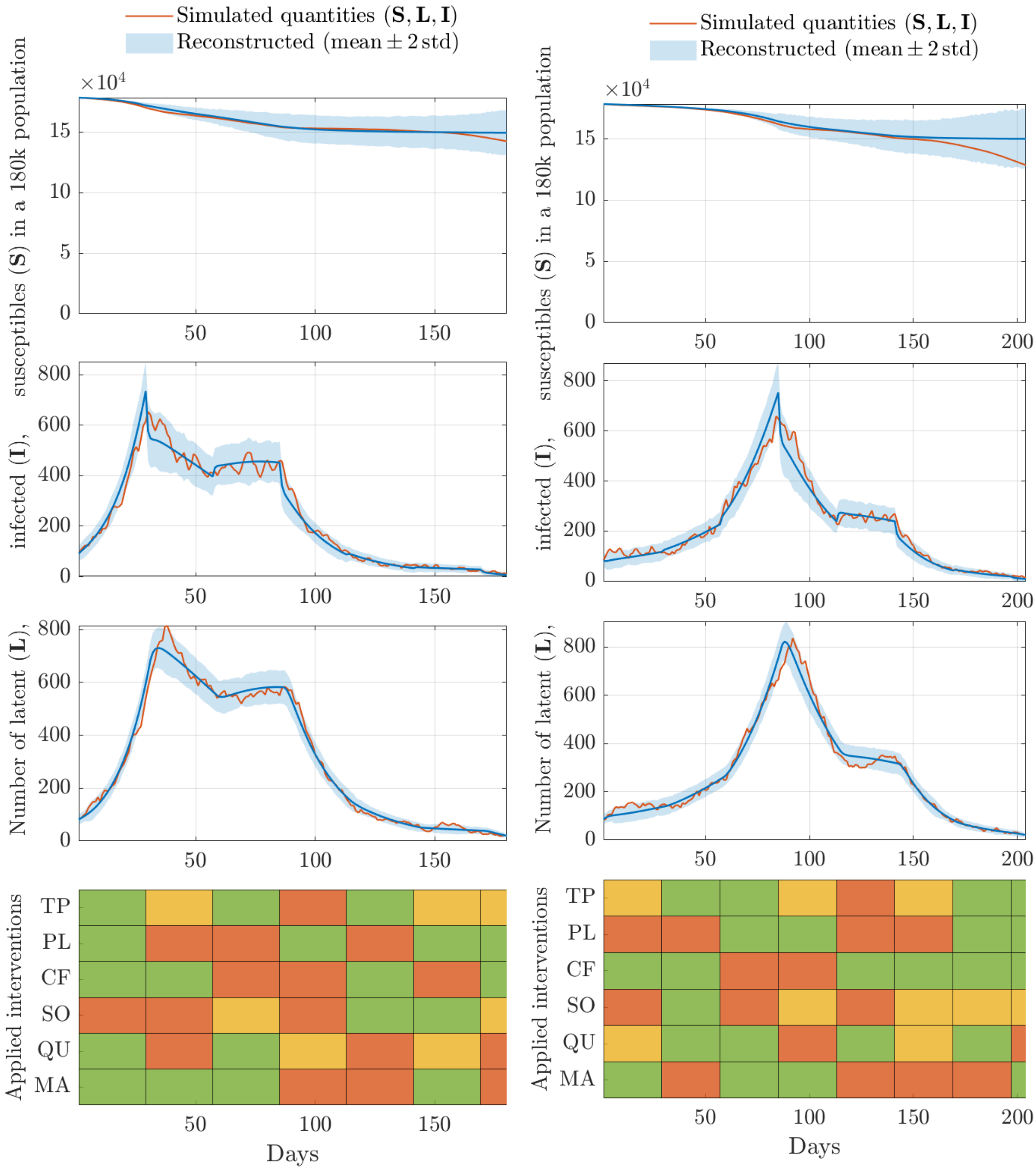

Figure 23: Estimated reconstruction error for two different outbreak scenarios simulated with two different sequence of interventions. The estimated 95% CI of the reconstruction is illustrated by the blue area.

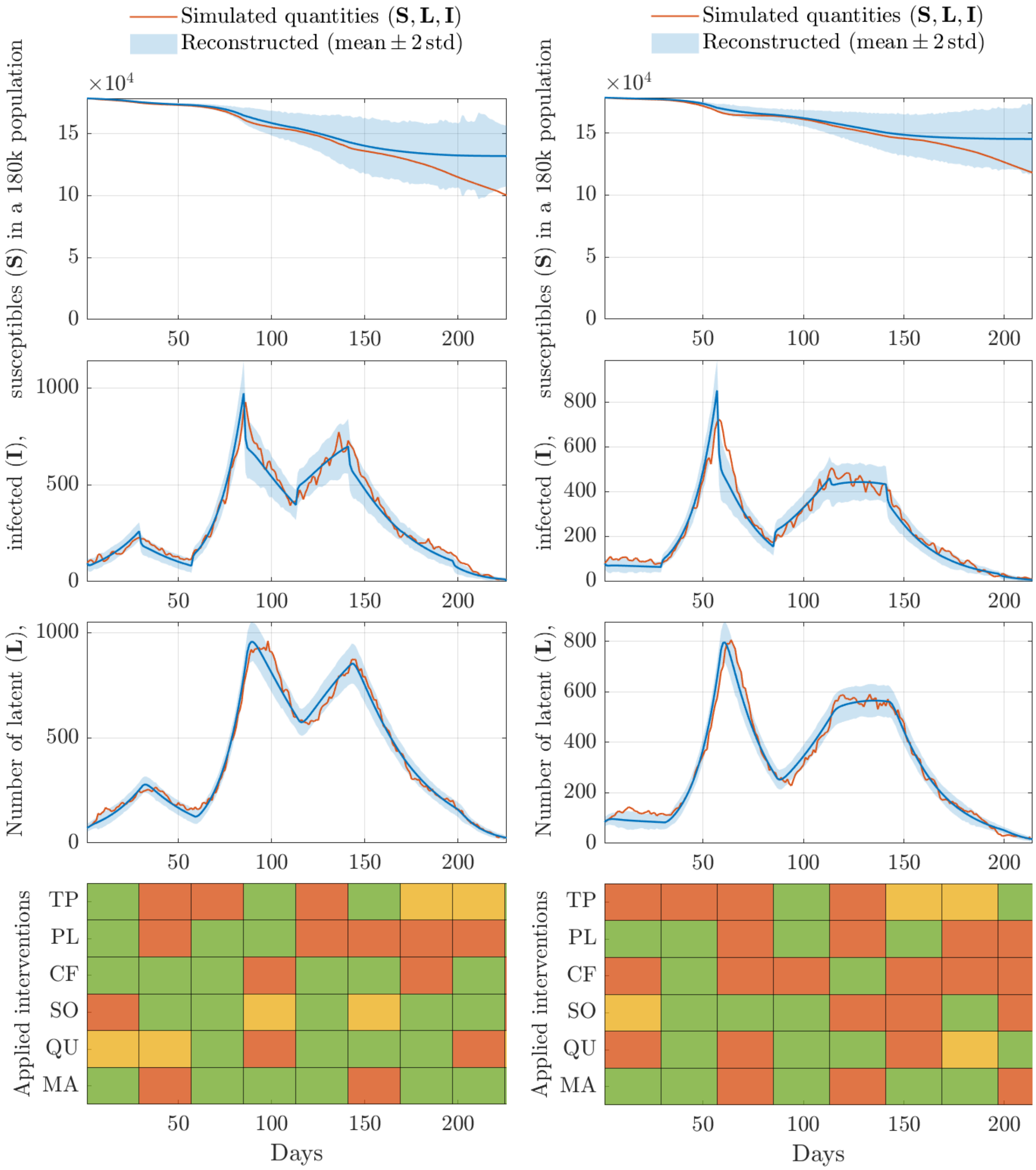

Figure 24: Estimated reconstruction error for two different outbreak scenarios simulated with two different sequence of interventions. The estimated 95% CI of the reconstruction is illustrated by the blue area.

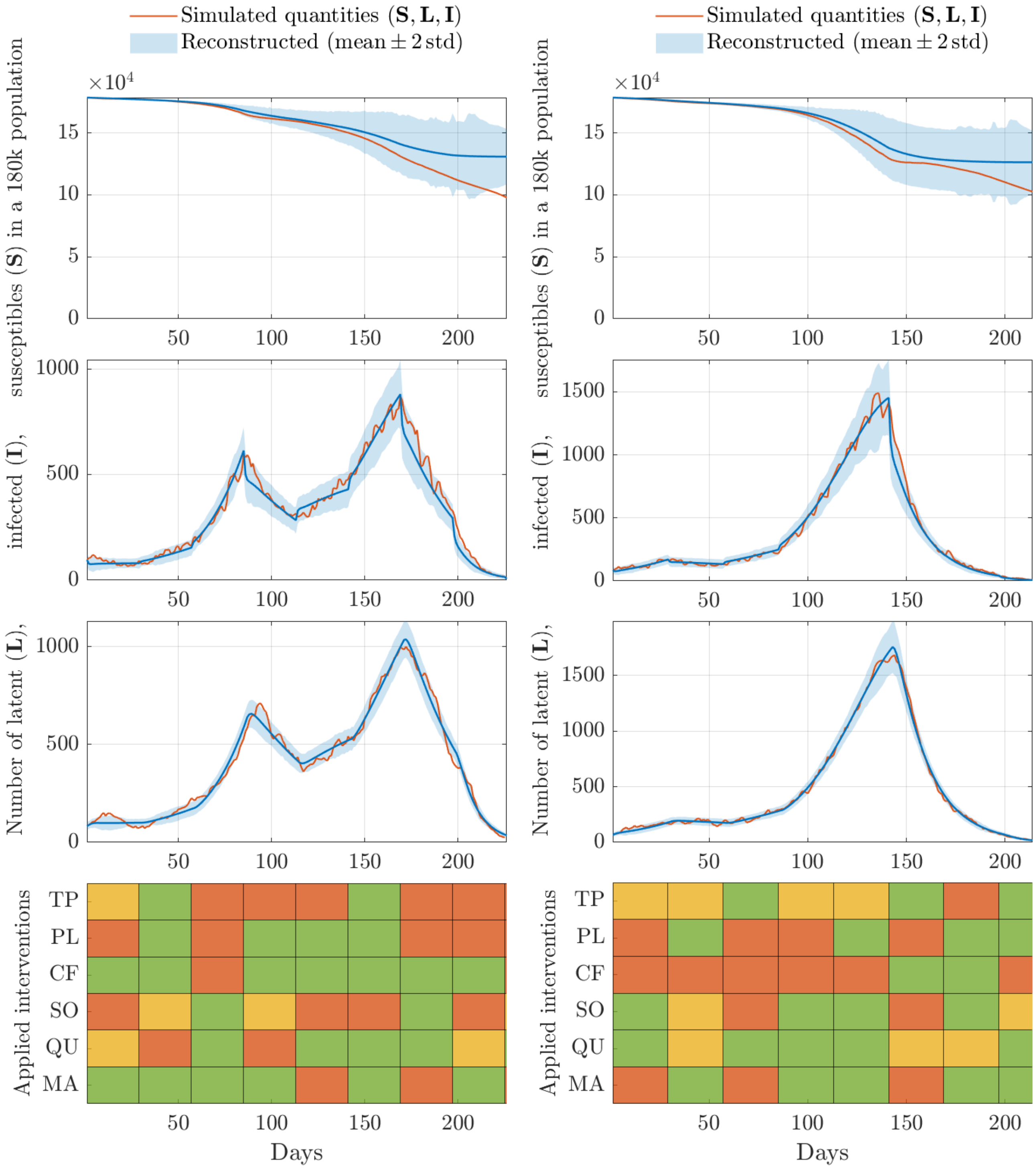

Figure 25: Estimated reconstruction error for two different outbreak scenarios simulated with two different sequence of interventions. The estimated 95% CI of the reconstruction is illustrated by the blue area.
