# Supplementary material for: Smart epidemic control: A hybrid model blending ODEs and agent-based simulations for optimal, real-world intervention planning
Source: PLoS Comput Biol. 2025 May 8;21(5):e1013028. doi: 10.1371/journal.pcbi.1013028 (PMC12061170; doi:10.1371/journal.pcbi.1013028)
Supplement: Attachment — Submitted filename: Responses_revisions.pdf [file pcbi.1013028.s003.pdf]

# List of Changes and Response to the Reviewers for the revised paper "Smart epidemic control: A hybrid model blending ODEs and agent-based simulations for optimal, real-world intervention planning" (Manuscript no. PCOMPBIOL-D-24-01801)

by

P. Polcz, I.Z. Reguly, K. Tornai, J. Juhász, S. Pongor, A. Csikász-Nagy, G. Szederkényi

First of all, we are grateful to the anonymous reviewers for the helpful and constructive comments and important observations.

Our answers are listed in the order of the comments and suggestions raised by the Reviewers. The section, equation, and citation numbers used in the responses are relevant to the revised version of the manuscript. The modifications are indicated with red color in the revised manuscript. The reference numbers of the revised manuscript are highlighted by red color in this response, e.g., Eq. (4), or Line 678 of the revised manuscript are referred to as (4) or Line 678.

## Contents

1 Review summary 2

2 Reviewer #1 2

3 Reviewer #2 7

4 Reviewer #3 12

5 ODE-based predictions compared to agent-based simulations 15

# 1 Review summary

**Comment 1** (Associate editor's comment). *All three reviewers made suggestions that entail substantial changes in the manuscript. I think that it is feasible to address these suggestions, which would lead to an improved paper. That said, some of these suggestions would require considerable additional work that may be excessive. Specifically, Reviewer #1 suggests modifying the model to another pathogen and comparing the results to COVID, and Reviewer #2 suggests comparing the proposed method with another control strategy. While those studies would indeed be interesting, and the authors are welcome to pursue them if they wish to, I don't think that they should be essential requirements for the acceptance of the paper.*

> ***Response.*** We sincerely thank the Editor for his understanding regarding the substantial effort required for a detailed quantitative evaluation of alternative control strategies or the development of additional numerical examples. We agree that such extensions, while significant, would exceed the time constraints for revision but also the scope of this manuscript.
>
> We are also grateful for not making these extensive revisions a requirement for possible acceptance. Based on the Editor's guidance, we have carefully revised the manuscript and addressed the reviewers' comments to the best of our ability, incorporating meaningful clarifications and improvements.

In Section 5, we visualize the results of a control simulation of Scenario A ("Flatten the curve"), which is also included in a supplementary document in the hope that this short "animation" gives a better insight for the reader, how the control algorithm actually works. The illustration may complement the answers to multiple reviewer comments.

# 2 Reviewer #1

*The authors prevent a hybrid ODE and agent-based model for determining what combination of interventions would be required to achieve a desired epidemic trajectory, and suggest that their approach could be adapted to other diseases.*

> ***Response.*** Thank you for the time and effort you have taken to review our manuscript, and the constructive comments/suggestions to improve our results. We have made a significant effort to address all the raised issues and comments.

**Comment 2.** *The model seems to be "fit for purpose" as demonstrated. However, I kept wondering what the advantage was of such a complex approach. Most of the interventions described can be fairly simply described in terms of impact on either the number of contacts or (nearly equivalently) the transmission rate (beta). If you need to reduce the number of infections by 30% to meet your target, can't you just choose (rather than simulate) the set of interventions that will get you very close to that? Relatedly, I would be curious to know how well just the ODE model performs on these tasks without including PanSim.*

> ***Response.*** We agree that the impact of the intervention on the number of contacts, separately for each measure, can be easily described. Similarly, we acknowledge that the stringency of interventions is often characterized in terms of their impact on the transmission rate ($\beta$).

However, we argue that assessing the combined impact of multiple concurrent measures is not straightforward, as their joint effects are often not straightforward to characterize. For instance, intensive testing and contact tracing may have little to no effect in the absence of even mild quarantine policies. The Oxford Stringency Index [1] was a notable attempt to quantify the cumulative impact of various measures, but it relies on predefined heuristics rather than direct estimation from data.

Our approach explicitly estimates the effect of each combination of interventions:

$$I = (\mathrm{TP}, \mathrm{PL}, \mathrm{CF}, \mathrm{SO}, \mathrm{QU}, \mathrm{MA}) \quad \in \quad \mathcal{I} = \{\mathrm{low}, \mathrm{high}\}^3 \times \{\mathrm{low}, \mathrm{medium}, \mathrm{high}\}^3 \tag{1}$$

by computing the mean ($\bar{\beta}$) and standard deviation ($\sigma_\beta$) of the transmission rate ($\beta$) based on synthetic data generated with PanSim. Consequently, when the goal is to reduce the maximum possible transmission rate (in a free population, $\beta_{\mathrm{max}}$) of infections by 30%, i.e., to achieve a rate

$$\beta_{\mathrm{d}} = 0.7 \cdot \beta_{\mathrm{max}}, \tag{2}$$

we obtain a set of feasible combination of measures:

$$\mathcal{I}_{30\%} = \big\{ I \in \mathcal{I} : \beta_{\mathrm{d}} \in \bar{\beta}(I) \pm \sigma_\beta(I) \big\}. \tag{3}$$

As the combination of measures achieving a given effect is not necessarily unique, this flexibility allows selecting the measures based on heuristics or specific objectives. For instance, criteria can be introduced to minimize changes in interventions across the intervening cycles. We admit that there are still many opportunities to refine and expand this methodology how to select the "currently best" NPI from $\mathcal{I}_{30\%}$.

*"I kept wondering what the advantage was of such a complex approach."*

Our approach is capable of tracking not only a constant target ($\beta_{\mathrm{d}} = 0.7$) but also a complex time-dependent trajectory. We also highlight that control is implemented within a feedback loop, meaning that predictions are recalculated at each planning cycle based on the latest estimated epidemic state. Due to the stochastic nature of the process, this may lead to dynamic adjustments, where an initially proposed intervention is later revised based on updated information.

**Illustrative Example:** Consider a scenario where, in October, intervention $I_1$ is expected to yield $\beta(I_1) = 0.7\,\beta_{\mathrm{max}}$, which appears sufficient to reach a state where, in November, a more lenient intervention $I_2$ with $\beta(I_2) = 0.9\,\beta_{\mathrm{max}}$ would be adequate to ensure a downward trend in infections before a major conference in December. However, an unforeseen event, such as a large festival in October, disrupts the expected control effect. Consequently, by the end of October, updated calculations reveal that the target cannot be achieved with $I_2$, necessitating a stricter intervention $I_3$ with $\beta(I_3) = 0.8\,\beta_{\mathrm{max}}$ in November to mitigate the risk and ensure the conference proceeds without disruptions.

Unlike a purely feedforward approach – where interventions would be precomputed based on initial estimates without adjusting to real-time changes – our feedback-based method enhances the robustness of the control algorithm. This robustness can be further improved by selecting a shorter intervention planning cycle (e.g., 7 days instead of one month). In this way, the actual state is estimated weekly, and both the prediction and the control are recalculated weekly.

One of the major questions throughout our work has been how to map the continuous-range scalar-valued control signal ($\beta$) to a set of practically applicable measures in a meaningful way? We found heuristic approaches [2]. Others estimated the effects of the distinct

interventions or a few combinations of interventions [3, 4] from the measured data of real epidemic outbreaks. In the current work, we followed [5] and generated virtual data using the georeferenced, age-stratified agent-based model PanSim.

*"I would be curious to know how well just the ODE model performs on these tasks without including PanSim."*

The control method we propose is essentially a predictive controller that utilizes a deterministic *ordinary difference equation* (ODE) as its prediction model. The computed control action is expressed in terms of the pathogen's transmission rate. Up to this point, our control approach closely resembles that of [6], where the computed transmission rates were not mapped to interventions but were instead directly *applied to* or *tested on* the ODE model itself.

In response to the reviewer's request, we added new illustration to the supplementary material S1 Text, in which the ODE-based predictions are compared to the simulation results of PanSim. The illustrations are also provided by the end of this response letter in Figures 5–12. In these figures, present a control simulation, during which the computed measures are recalculated monthly according to the actual outcome of the simulated process (which may be different from that predicted one month before). In each figure, we illustrate the target curve (red), the predicted curve (for the "current future"), the simulation result and its reconstruction.

**Comment 3.** *The authors mention* "it is straightforward to modify the parameters and fit the ABM to a different pathogen" *(p. 7). This seems unlikely to me for a couple reasons. First, this is almost never as easy as it seems! Second, the code seems to be written in C++ and Matlab, which are both suitable choices for high-performance simulations, but neither of which are that familiar to the majority of epidemiologists (though the authors are thanked for making their code public, and it seems well-written). The authors don't discuss how long the model takes to run or what compute resources are required, but I imagine it is significant if a GPU implementation is warranted. Finally, if the model is* "straightforward to modify", *the authors are encouraged to modify it to another pathogen (e.g. mpox, or another non-respiratory pathogen of relevance to their work) and compare-and-contrast the findings with COVID.*

***Response.*** Thank you for this clarifying comment. We acknowledge that adapting an ABM to fundamentally different pathogens (e.g., non-respiratory transmission) is not straightforward. We revised the quoted statement at Line 164 and clarified that the model is flexible primarily for respiratory pathogens like influenza virus, respiratory syncytial virus (RSV), or measles virus, which are transmitted through aerosols or respiratory droplets. As long as a new pathogen has a similarly structured disease progression, the transition probabilities, time constants and other parameters can be updated, even without touching C++/Matlab code. The key parameters associated with a pathogen described in [7], are stored in JSON (for C++) and spreadsheet files (for Matlab). Indeed, in Scenario **F** (Fig. 5), we evaluate our approach on the Omicron variant, which is substantially different from the original wildtype in these parameters.

Although, with reference to the Editor's Comment 1, conducting a complete study with a different respiratory pathogen is beyond the scope of this work. However, we highlight this as a future research direction.

> *"The authors don't discuss how long the model takes to run or what compute resources are required, but I imagine it is significant if a GPU implementation is warranted."*
>
> PanSim was designed to support large numbers of simulations executing both on personal computers (PC) or computer clusters. In our present work, all simulations were performed on a relatively old mid-range architecture, a desktop PC with Intel Core i5-4590 CPU at 3.30 GHz with 32 GBs of DDR3 RAM at 1333 MHz and a GeForce GTX 750 Ti GPU with 2GBs of GDDR5 integrated memory. On this architecture, simulating the spread in a city like Szeged with 180k agents and 81k locations in a 210 days (30 week) long time frame takes approximately 69 seconds. Therefore, generating 1000 simulations for constructing the lookup table requires about 19 hours. We remark that PanSim does not require a GPU necessarily. If the same simulation is executed on a laptop PC with Intel Core i7-4710MQ CPU at 2.50 GHz with 16 GBs of DDR3 RAM at 1600 MHz (without using a GPU), the execution time is about 500 seconds. We have added the main simulation conditions and parameters to the revised version of the paper. More implementation details of PanSim are discussed in our earlier papers [7, 8]. Moreover, we added a section in the supplementary material S1 Text with the hardware information above.

**Comment 4.** *p. 5: 10 min is a very small timestep – how much do results differ if the timestep were an hour or even a day?*

> ***Response.*** Thank you for this observation. We chose the 10-minute timestep in order to capture short-duration activities and infection dynamics, as potentially risky exposure times in crowded environments can be as short as 15-20 minutes. Therefore, increasing the timestep to an hour or a day would reduce the descriptive power and accuracy of the model.

**Comment 5.** *p. 5: 10% forecast accuracy 3 weeks in advance isn't bad. However, given that hospitalizations usually lag infections, often much simpler measures (such as test positivity rate) can also get good performance predicting hospitalizations.*

> ***Response.*** Thank you for your comment. We would like to argue that the delay in hospitalization data does not necessarily degrade the quality of the reconstruction. Since, the delay from infection to hospitalization is implicitly modeled through the transition rates (i.e., time constants $\tau_\mathrm{I}, \tau_\mathrm{P}, \tau_\mathrm{L}$[1/days]) of compartmental transitions $\mathbf{L} \to \mathbf{P}$, $\mathbf{P} \to \mathbf{I}$, and $\mathbf{I} \to \mathbf{H}$. We acknowledge that reconstruction could benefit from incorporating additional indicators, such as test positivity rates, which are already integrated into PanSim for modeling quarantine policies. However, during the first two years of the COVID-19 pandemic in Hungary, hospital data proved to be the most reliable among the available time series [4]. From 2022 onward, the introduction of extensive nationwide wastewater analysis significantly enhanced the accuracy and performance of epidemic reconstruction efforts [9]. While the current approach uses hospital data due to its reliability in practice, test positivity rates and wastewater data could also be integrated. As noted in the manuscript, the reconstruction is a simplified version of wastewater-based methods and is sufficient for control, though incorporating additional data sources would probably further improve performance. See Comment in Line 172.

**Comment 6.** *p. 6:* "Secondly, our study focuses on managing the epidemic over a period of about six months, assuming that the initial virus strain does not mutate during this time. Therefore, it is reasonable to assume that the effects of the non-pharmaceutical interventions are time-invariant." *I think time-invariance is a reasonable assumption, but I don't think this is why. The impact of, say, school closures, changes the number of contact events that occur. The effect of reducing contacts should be time-invariant regardless of* "mutations" *(and, indeed, new variants certainly can arise within 6 months).*

***Response.*** We assumed that the time coefficients ($\tau_\bullet$) and probabilities ($p_\bullet$, $q_{\mathrm{A}}$) of the diseases progression are constant indifferently of the mutations. However, we agree that excluding the possibility of mutations within 6 month is not reasonable.

In the revised paper, we allow for the possibility of new mutations arising during the six-month period. However, for each mutant or variant, we assume that the maximum transmission rate, $\beta_{\max}$, remains time-invariant. We also acknowledge that what we are effectively controlling are social mobility (through restrictions) and the relative transmission rate (through measures such as masking). These interventions scale the free spread transmission rate by a given factor $u(I)$, which depends on the interventions $I$ applied.

In a previous paper of our research group [8], we demonstrated through simulations that $u(I)$ is a consistent scaling factor across all stages of an outbreak. Thus, a combination of interventions introduced at the beginning or the middle of an outbreak is expected to result in a similar transmission rate $\beta = u(I) \cdot \beta_{\max}$.

In the manuscript, we added a new assumption starting at Line 248.

**Comment 7.** *p. 7:* "Then, the LUT is filled in with the averages of the resulting transmission rates." *Why not fit a Gaussian process emulator or similar? Wouldn't this be more efficient, more accurate, or both?*

***Response.*** A non-pharmacological intervention (NPI) is a combination of measures described as a tuple of discrete values

$$I = (\mathrm{TP}, \mathrm{PL}, \mathrm{CF}, \mathrm{SO}, \mathrm{QU}, \mathrm{MA}) \quad \in \quad \mathcal{I} = \{\mathrm{low}, \mathrm{high}\}^3 \times \{\mathrm{low}, \mathrm{medium}, \mathrm{high}\}^3 \tag{4}$$

where TP, ..., MA are described in Table 1 of the manuscript.

Each combination $I$ of measures $(\mathrm{TP}, \mathrm{PL}, \mathrm{CF}, \mathrm{SO}, \mathrm{QU}, \mathrm{MA})$ results in a transmission rate $\beta(I) \in (0, 1]$. Due to the stochastic nature of the epidemic process, $\beta(I)$ is inherently a random variable, which is estimated by a Gaussian distribution. Therefore, it is natural to estimate the mapping $I \mapsto \mathcal{N}\big(\bar{\beta}(I), \sigma^2_\beta(I)\big)$, which can describe either by a Gaussian process (GP) or a lookup table $\mathrm{LUT} : I \mapsto \big(\bar{\beta}(I), \sigma_\beta(I)\big)$.

In our case, we have sufficient measurements for all possible $I \in \mathcal{I}$ to compute reliable statistics $\big(\bar{\beta}(I), \sigma^2_\beta(I)\big)$ directly. In our current database, we have at least 467 days simulated for a single combination of measures $I$. In average, we computed about 850 days for a single NPI. The Gaussian processes would be beneficial if we were unable to evaluate the statistics on $I \to \beta(I)$ for each $I \in \mathcal{I}$ separately.

On the other hand, our control algorithm requires evaluating the inverse mapping $\beta \to I$. If we had used a GP, we would still need to evaluate the GP at every $I$ to obtain expected values and variances, and then identify the $I$ giving the closest $\beta(I)$ to the desired $\beta_{\mathrm{d}}$. This process is essentially equivalent to using a GP to fill in a lookup table, which does not

justify the added complexity in this context.

Alternatively, we could estimate the inverse mapping $\beta \to I$ directly using a Gaussian process classifier. However, this is more challenging to model due to the inherently non-surjective nature of the $\beta \to I$ mapping, where multiple $I$ values may correspond to the same $\beta$. Additionally, a single GP classifier with 216 distinct classes (one for each $I$) would face significant difficulties due to the high number of classes. Splitting the task into six GPs for the six measures would neglect the strong dependencies between the measures, leading to additional modeling gaps.

The results suggested that directly constructing the LUT provides sufficient performance for our needs without introducing unnecessary complexity. The "cheap" simulation also justify to compute direct statistics for every combination of measures.

**Comment 8.** *p. 10:* "One possible monthly sequence of NPIs is presented" – *sorry if I missed it, but what happens when the same outcome can be achieved in multiple different ways (e.g., school closures or curfew, but without needing both)?*

***Response.*** We agree that the combination of interventions achieving a given effect is not unique. However, this flexibility is not a limitation; rather, it allows for selecting combinations based on specific objectives. For instance, criteria can be introduced to minimize changes in interventions across the intervening cycles. Other promising directions for future work can be incorporating socio-economic considerations, though they may introduce performance trade-offs.

We added two new paragraphs at Lines 297 and 741 to discuss these aspects.

## 3 Reviewer #2

*The authors present a study on model predictive control (MPC) applied to the simulated phases of the COVID-19 pandemic in Szeged. They employ a highly detailed COVID-19 agent-based model of Szeged, previously published in PLOS Computational Biology. This model is used to both estimate unknown variables and evaluate the impact of a prescribed set of non-pharmacological interventions (NPIs).*

*The primary limitation of the study is the lack of real-world data to validate the control algorithm. However, this is justifiable given that real-world testing would require waiting for a future pandemic, which is impractical.*

*Overall, the manuscript is well-written, and the approach is both interesting and innovative. Nevertheless, I believe the study would be further strengthened by addressing the following points:*

***Response.*** We would like to sincerely thank Reviewer 2 for his/her thoughtful and constructive feedback. We greatly appreciate the positive comments regarding the manuscript and the approach. We also acknowledge the Reviewer's concern regarding the lack of real-world data to validate the control algorithm, and we agree that it is a limitation that is inherently tied to the nature of pandemic modeling. We have addressed all the points raised and made the necessary revisions to further strengthen the study.

**Comment 9.** *Quantify the accuracy of the state reconstruction method.*

***Response.*** Thank you for the constructive comment as it led us to address new sub-directions of this project.

To quantify the accuracy of the state reconstruction, we built a synthetic data set from 1000 simulation records. This set comprises data frames of about 237$k$ simulated days in total. To estimate the uncertainty of the reconstructed value for **L** (the number of infected in the latent phase), we approximated separate statistics for multiple admissible intervals for **L** (according to the moving window principle). We repeated this study for compartments **P**, **A**, **I**, and **S**. The distribution of the 237k samples and the expected state estimation error for the different ranges of **I**, **P**, **A**, **I**, and **S** are illustrated in Figure 1. Figure 2 visualizes the reconstruction accuracy for two different outbreaks simulated.

The uncertainty analysis results are documented in brief in Supplement S1, which is referred in the main text at Line 704.

We note that having expert knowledge on the uncertainty of the model parameters, one can adopted the more sophisticated uncertainty analysis approach from our previous work [4], in which we computed the standard deviations of the reconstructed states by solving a recursion equation of the (approximation) mean and variance dynamics of the stochastic state-space model. As a future work, we will asses whether it is reasonable to estimate the (possibly joint) distribution of the model parameters $(\tau_\bullet, p_\bullet, q_{\text{A}}, q_{\text{H}})$ from simulation data, which would make possible to formally characterize the reconstruction error.

**Comment 10.** *Quantify the accuracy of the lookup table method.*

***Response.*** Thank you for the suggestion. Accordingly we provided additional details in supplementary documents, which we presented in brief in this response letter too.

Each combination $I$ of measures $(\text{TP}, \text{PL}, \text{CF}, \text{SO}, \text{QU}, \text{MA})$ results in a (probabilistic) transmission rate $\beta(I) \in (0, 1]$, which is estimated by a Gaussian distribution $N\big(\bar{\beta}(I), \sigma_\beta^2(I)\big)$. Considering (again) the 237k days-large simulation record, we compute the mean and standard deviation (Std) of the resulting transmission rates separately for each of the 216 combination of measures. These values are provided in a supplementary spreadsheet file (Supplement S2).

Figures 3 and 4 (also included in Supplement S1) illustrate the uncertainty of the LUT. We note that the "medium" and "low" values for the quarantine policy (QU) resulted in a similar value for mean and Std of the transmission rate. Therefore, solely for visualization purposes, those case with "medium" QU policy are not included in Figures 3 and 4.

In Line 723, we refer to Supplement S2 and the appropriate section of Supplement S1.

**Comment 11.** *Comparison with alternative approaches: If feasible, comparing the proposed method with another control strategy could demonstrate its relative strengths or limitations.*

***Response.*** We have extended the DISCUSSION section by comparing our methodology with the most closely related approaches. For this, we have found and added several new references to the bibliography. We have also extended the literature review in the

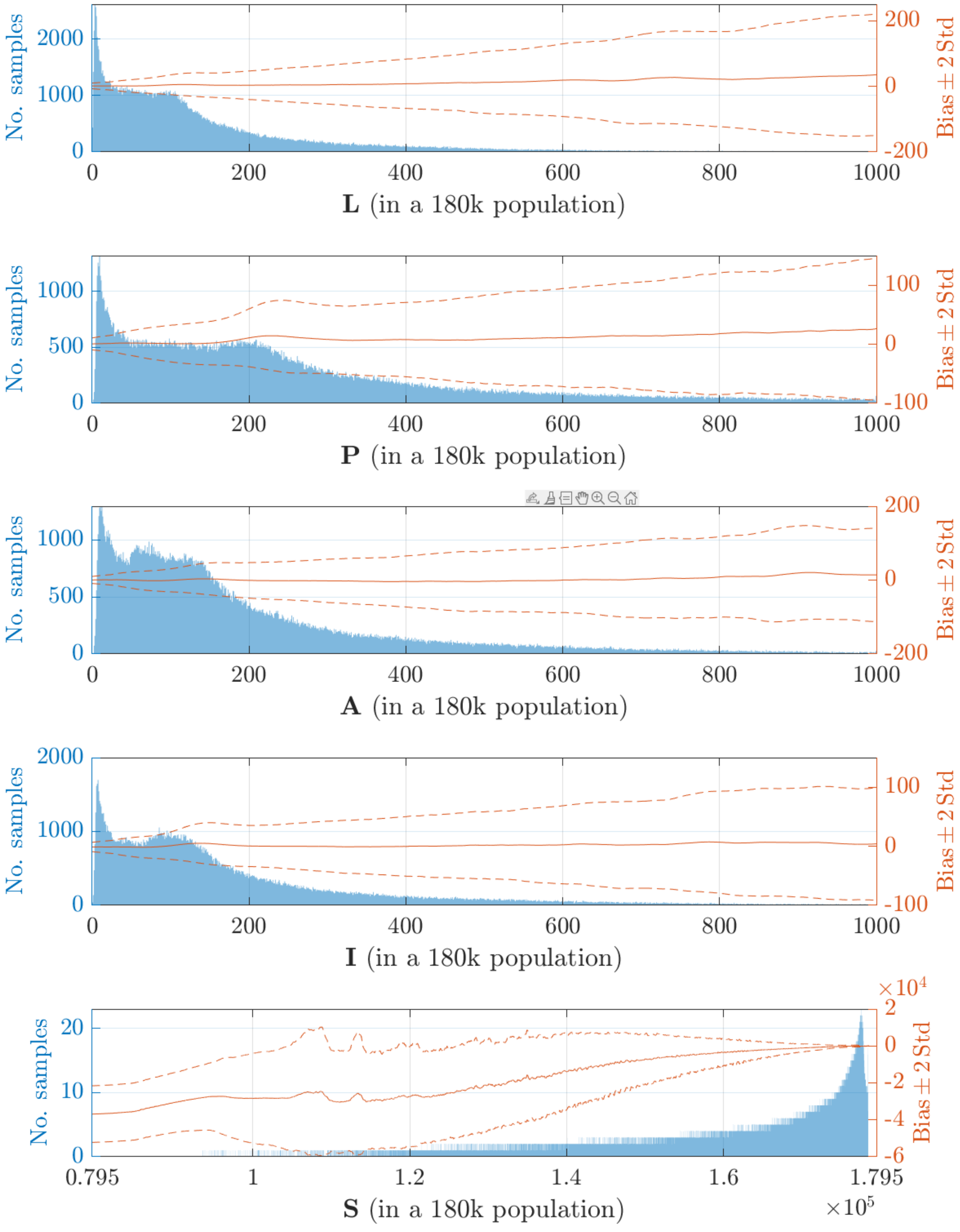


Figure 1: Distribution of samples and the uncertainty of the reconstruction. The histogram shows, how the 236,935 samples are distributed with respect to the distinct values of the simulated quantities **L**, **P**, **A**, **I**, **S** (left axes). The solid lines illustrates the average bias error of the reconstruction, whereas, the pairs of dashed lines bound the 95% CI of the bias error (right axes). E.g., the third plot illustrates that the reconstruction of **A** is about 1% biased and the relative error is less than 12%.

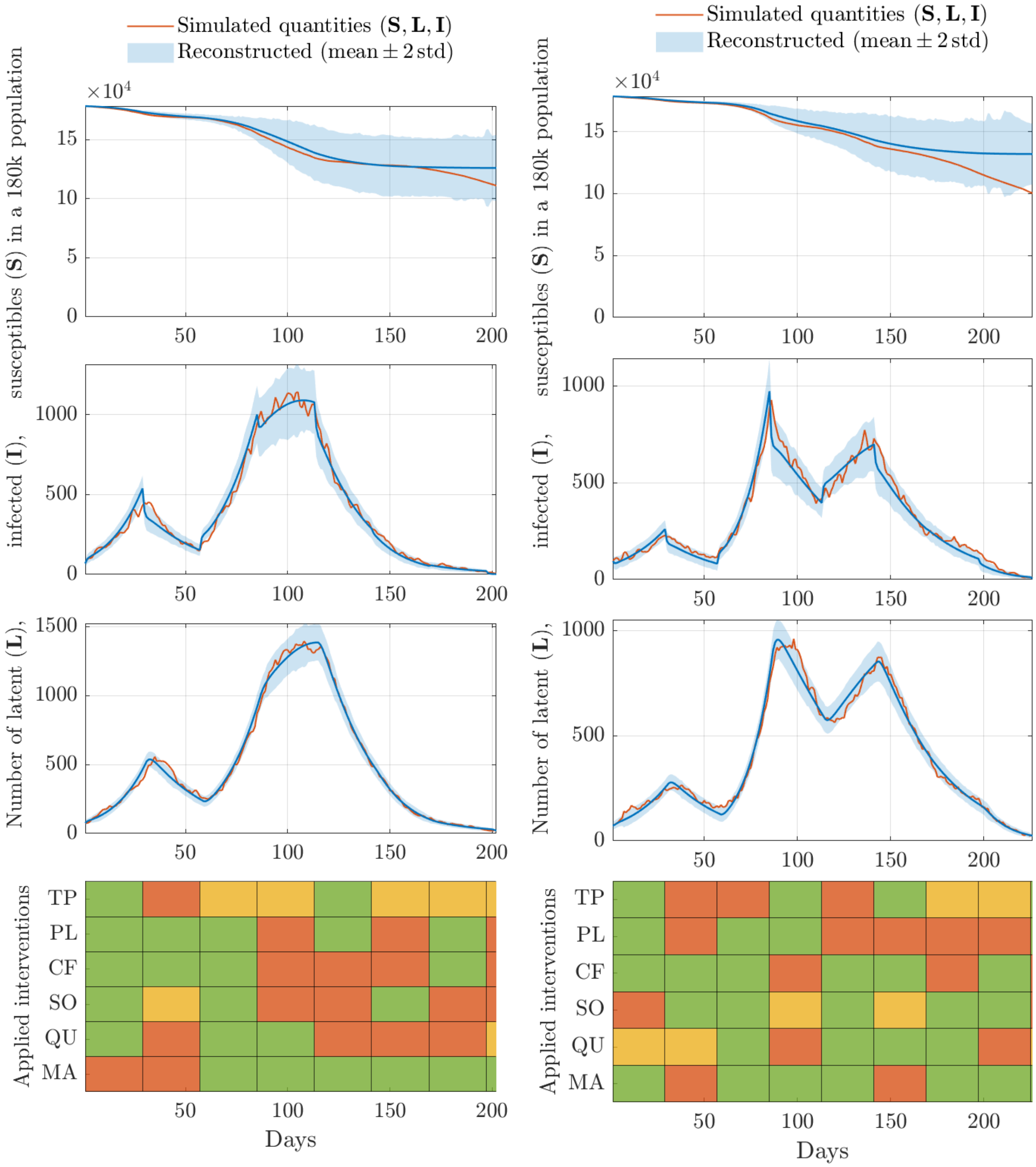


Figure 2: Estimated reconstruction error for two different outbreak scenarios simulated with two different sequence of interventions. The estimated 95% CI of the reconstruction is illustrated by the blue area.

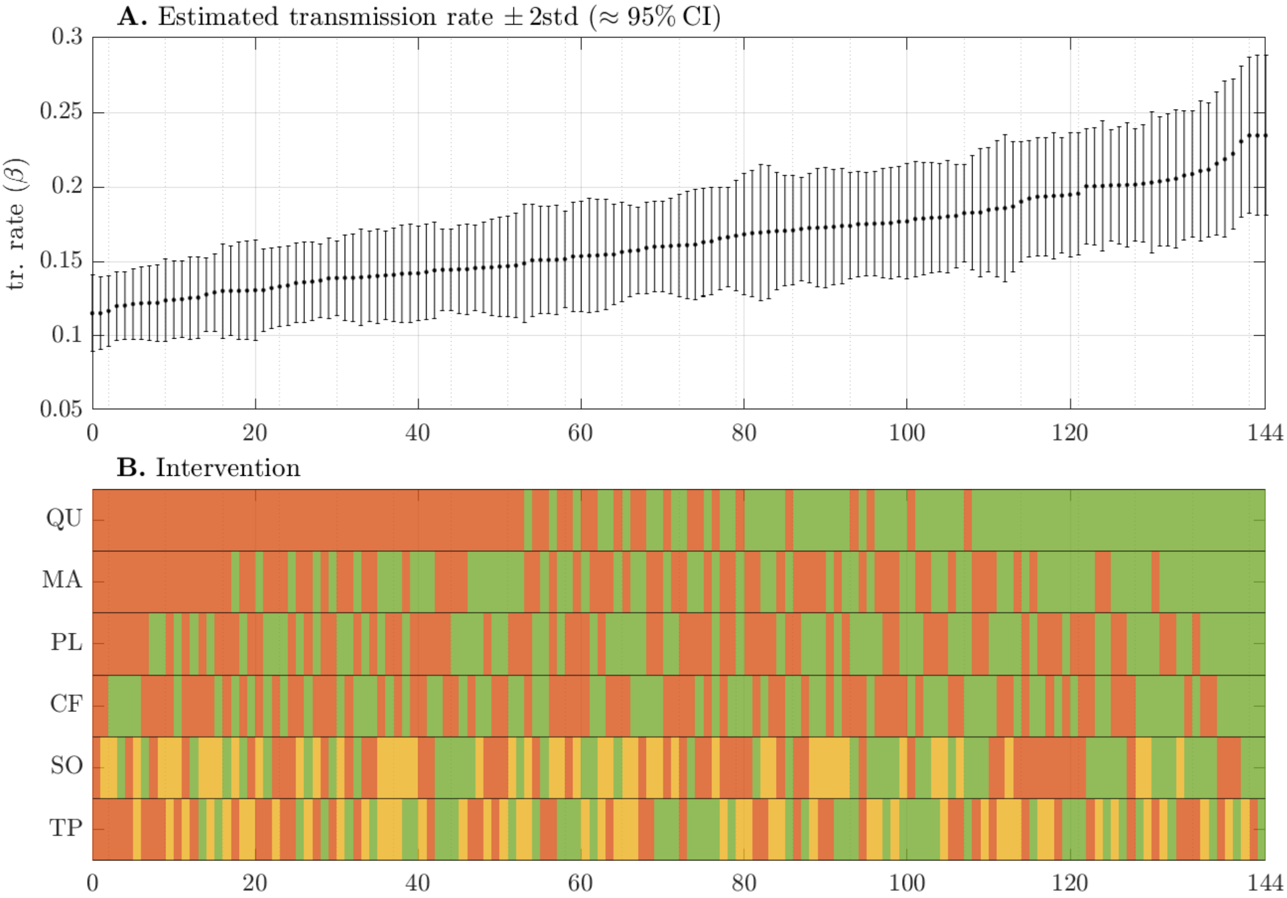


Figure 3: Mean and variance of the estimated transmission rates $\beta$ for the different combination of measures ordered with respect to the obtained mean value for $\beta$.

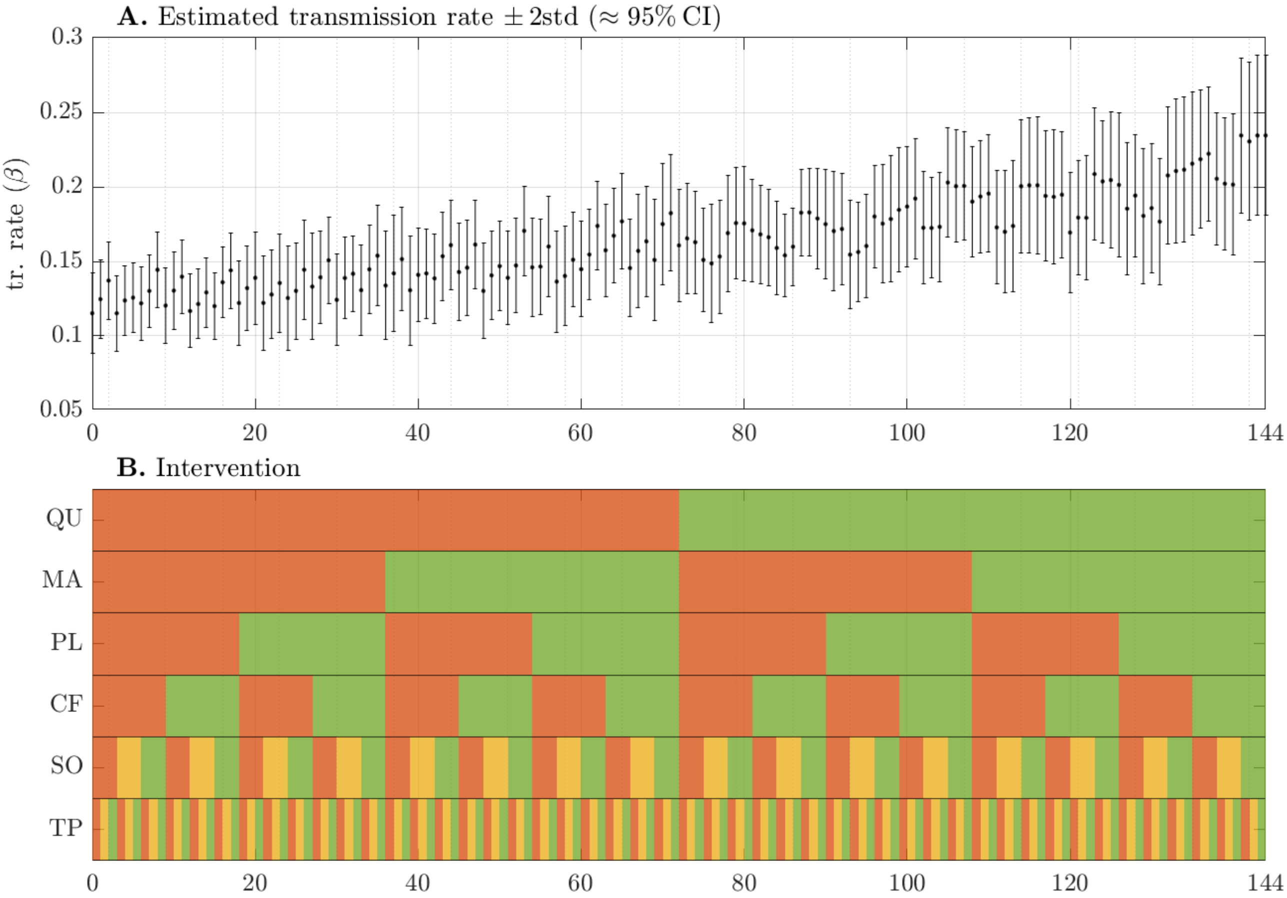


Figure 4: Mean and variance of the estimated transmission rates $\beta$ for the different combination of measures ordered with respect to the stringency of the measures.

INTRODUCTION with the new references.
(We also refer to the response given to Comment 14.)

**Comment 12.** *Minor Suggestions:*

1. *Line 129 (in the first version of the manuscript): Remove the extra period.*
2. *Figures: Improve figure captions to make them more detailed and enable independent interpretation without referring to the main text.*
3. *Line 409: In silico should be italicized.*
4. *Line 606: Rephrase* "In the authors' experience" *to make it more formal and objective.*
5. *Line 672: The phrase* "Somewhat, we want to simulate a real epidemic event" *could be revised.*
6. *Consider reducing the use of abbreviations, as the text contains many that might hinder readability for a broader audience.*

***Response.*** Thank you for the time and effort you have taken to review our manuscript, and give constructive comments/suggestions to improve our results. We did our best to address all your comments and suggestions.

# 4 Reviewer #3

*This paper addresses the issue of how to optimally choose interventions to control an epidemic. Specifically, they consider a detailed agent-based model (ABM) which contains extensive details of the spread of the pathogen as well as the effect of potential interventions. The full space of policy choices is considered too complex and computationally expensive to explore using the agent-based model. To solve this problem, they pair the complex model with a simple ODE model where the effect of interventions can be extensively analysed using model predictive control (MPC) strategies.*

**Comment 13.** *The exact contribution of this paper was unclear with the message often getting lost amongst the details. The paper needs to be dramatically shortened (i.e. 10 pages as opposed to 26), with the novel contribution and methodology being clearly articulated, and unnecessary details removed to Supplementary Materials. For example, whilst the paper is about how to choose policy, it is not a policy paper discussing what policies should be used under which circumstances, rather it is a methodology paper. Therefore a detailed examination of scenario A, contrasting the results with other methodologies and developing a detailed understanding of why the results were as they were, would be far more impactful than a list of scenarios A-G.*

***Response.*** We think that the main contribution of the paper is the more realistic and practical nature of planning measures in a feedback configuration. Firstly, we can take into consideration a wider range of measures than what we found in the related literature. Secondly, the manipulable inputs (i.e., the possible levels of measures) are discrete valued which is also closer to practice. Thirdly, the change between measures applied during consecutive planning periods is also minimized. These features are the result of a non-trivial novel control design procedure which is able to map combinations of interventions to the

desired level of transmission rate. Therefore, the computation results can be directly given to epidemic experts and decision makers for consideration. Finally, we would emphasize the high performance of the agent-based simulation compared to other solutions, allowing a large number of simulation runs and thus the exhaustive testing of possibilities. We have clarified the contributions in the ABSTRACT, INTRODUCTION and DISCUSSION sections.

**Comment 14.** *The idea of having a simple surrogate model which is paired with an expensive detailed model is not novel. There is an extensive literature on using emulators in this way (often as part of a calibration method). The methodology of this paper needs to be compared to these established methods, with its advantages/disadvantages examined.*

***Response.*** We are grateful for the helpful suggestion. As the Reviewer suggested, we have revisited the literature that focuses specifically on surrogate modelling of agent-based simulators for control purposes. We found six further relevant contributions [2, 5, 10, 11, 12, 13], which are briefly described in INTRODUCTION. We found that three of these contributions are comparable with our framework (others consider learning-based surrogates or not used directly in control design). In DISCUSSION (Lines 523–557), we compare our framework both with [2, 12] and a recent result of Fonseca et al. [13] in terms of methodology, applicability, efficiency, and the underlying agent-based model.

One of the early works to recognize the potential of optimization-based control for agent-based models (ABMs) is presented by An et al. [5]. While we do not claim it was the first, this paper probably introduced the concept to a broader research community. It proposed a novel framework for optimizing and controlling ABMs by treating them as surrogates for real-world systems. By approximating ABM behavior with more mathematically tractable models, such as differential equations, the study laid the groundwork for integrating established optimization and control techniques into ABM-based decision-making.

The works of Niemann et al. [2, 12] address the challenges of predictive control design for agent-based epidemic models using surrogate modeling and multilevel optimization techniques. In [2], Koopman-based surrogate models are built to approximate the dynamics of non-deterministic ABMs efficiently, enabling the solution of multi-objective optimal control problems. Although they do not explicitly name it as such, their approach essentially constructs a model predictive controller (MPC) for a simplified agent-based epidemic model using a data-driven surrogate model. In [12], a heterogeneous multi-level optimization framework is proposed, combining detailed ABMs with coarse ODE models to improve computational efficiency in policy design while preserving essential epidemic dynamics.

**Comment 15.** *The paper talks about using an ODE model, but from my understanding of the equations in the methodology it is a discrete time compartmental model not an ODE model. If this is the case, then it should be described as such throughout.*

***Response.*** Thank you for pointing this out. You are right that the model is indeed a discrete-time nonlinear difference equation derived from an underlying ODE. However, within the field, it is common practice to refer to such models as ODEs because they originate from ordinary differential equations, even though their implementation operates in discrete time. The acronym ODE is sometimes also expanded as *"ordinary difference equation"*, as it is introduced in Line 26.

**Comment 16.** *One aspect of the epidemic which was key in 2021 was it's age stratified nature (e.g. see positivity rates in the UK data which could be different by a factor of over 10 between the age groups). With vaccination rates being vastly different between age groups, along with certain NPIs affecting different age groups (i.e. school closures), I don't see how a non-age stratified model can be an appropriate emulator of the full system. Explaining how the proposed methodology handles these situations is important to evaluate the validity of the approach.*

***Response.*** Thank you for your comment. The agent-based model underlying our framework is age-stratified and incorporates age-specific parameters, as demonstrated in Fig. 4 of our earlier PLoS CB publication [7]. This ensures that the dynamics of infection, including age-dependent positivity rates and vaccination coverage, are appropriately captured in the detailed simulation.

While the current control model does not include explicit age stratification, it is worth noting that the methodology can be extended to include age-stratified control objectives. PanSim can be adapted to generate age-specific outputs, enabling the optimization framework to account for the differential effects of NPIs across various age groups. Implementing such an extension is technically feasible; however, it is questionable whether it is worth the additional complexity and effort given the broader goals of this study.

We clarified this in Section PanSim, a detailed agent-based simulator (between Lines 140–168).

**Comment 17.** *From my understanding of the methodology, the parameters ($\rho$) of the simplified model are estimated once for the whole simulation. Given that the ABM is a much more rich model, my expectation would be that they would vary over time depending upon changes in the ABM states (e.g. vaccination rates, closures of specific types of institutions), which I expect this would give a better fit of the short-term dynamics within each policy period. The paper needs to examine the effect of only having a single set of model parameters.*

***Response.*** It is reasonable to assume that the average residence times $\tau_{\mathrm{L}}$, $\tau_{\mathrm{P}}$, $\tau_{\mathrm{I}}$, $\tau_{\mathrm{A}}$ in compartments **L**, **P**, **I**, **A**, the probability of symptomatic infection ($p_{\mathrm{I}}$), and the relative transmission rate of asymptomatic individuals ($q_{\mathrm{A}}$) depend primarily on the virulence and infectiousness of the pathogen, making them relatively invariant during the dominance of a single variant.

On the other hand, parameters such as the average length of hospitalization ($\tau_{\mathrm{H}}$), the relative infectiousness of hospitalized patients ($q_{\mathrm{H}}$), the probability of hospitalization ($p_{\mathrm{H}}$), and the probability of fatal outcomes ($p_{\mathrm{D}}$) could indeed be influenced by external factors like hospitalization policies or masking rules in hospitals. However, in this study, these factors are not varied. For instance, masking rules (MA) in both *low* and *high* policy levels always assume mask-wearing in hospital settings. Thus, while acknowledging their potential variability, we consider $\tau_{\mathrm{H}}$, $p_{\mathrm{H}}$, $p_{\mathrm{D}}$, and $q_{\mathrm{H}}$ to remain constant during the dominance of a single virus variant.

We added a short remark in the manuscript in Section Assumptions in Line 248;

**Comment 18.** *Whilst a link to a Github repository for the underlying ABM was provided, I could not find the code related to the work presented in this paper.*

***Response.*** Thank you for the observation. Indeed the Matlab implementation were uploaded to a different branch of the repository. Control-related code files are now uploaded to the main branch.

# 5 ODE-based predictions compared to agent-based simulations

Throughout Figures 5–12 (starting from the following page), we present a control simulation, during which the computed measures are updated monthly according to the actual outcome of the simulated process. In each figure, we illustrate the target curve (red), the predicted curve (for the "current future"), the simulation result and its reconstruction.

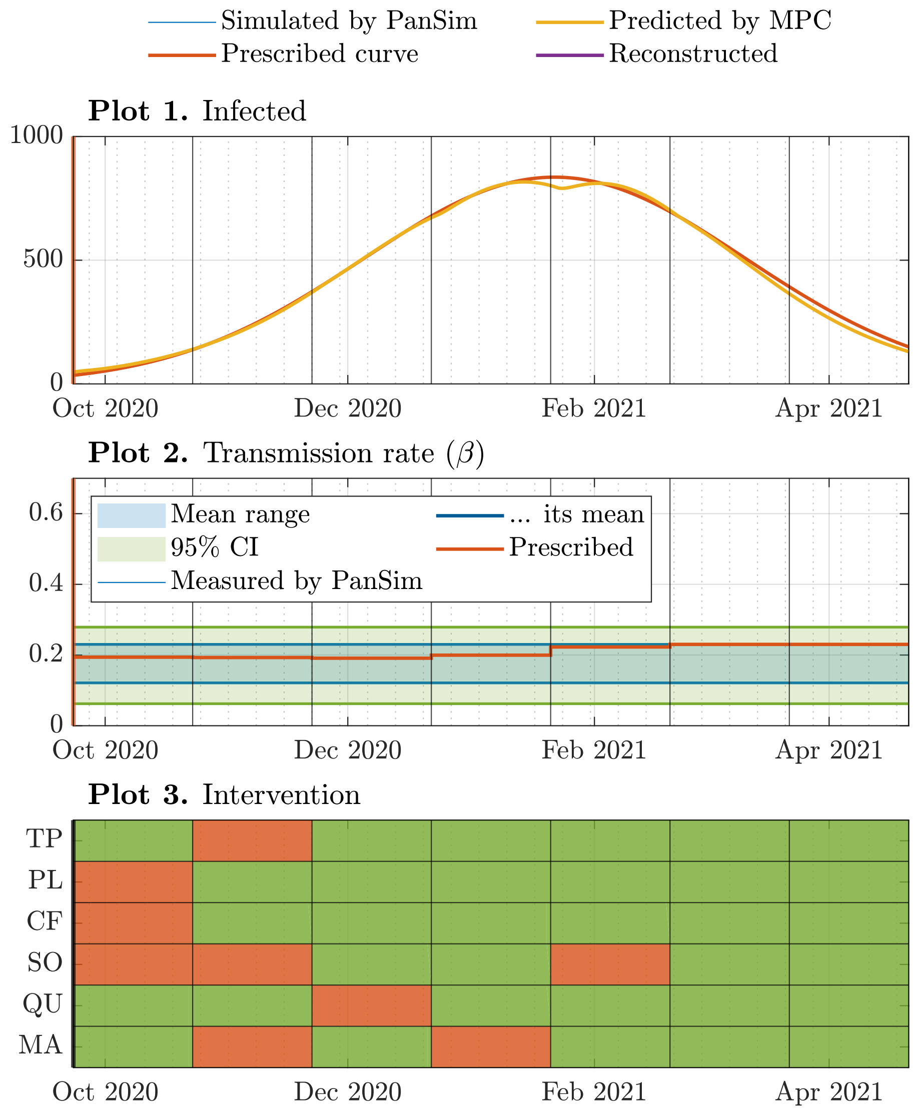


**Figure 5. Control simulation before the first intervention planing cycle (month).** The vertical red line illustrates the current time of the simulation, which is now at **day** 0. The red bell-shaped curve illustrates the prescribed trajectory for the infected people with symptoms in the main phase of the disease (**I**). The yellow curve, which slightly follows the reference curve is the trajectory for **I** predicted by the discrete-time ODE (i.e., the recursion equation) (2). In Plot 2. the red staircase function illustrates the necessary transmission rate $\beta$ to achieve the prescribed curve for **I**. The blue area in Plot 2 highlights the range of the computed mean values for $\beta$ for the different interventions, whereas, the green area illustrates the range of the mean $\pm$Std computed for the different interventions. Plot 3 illustrates the stringency (low: green, medium: yellow, high: red) planned for the six independent measures summarized in Table 1 of the manuscript.

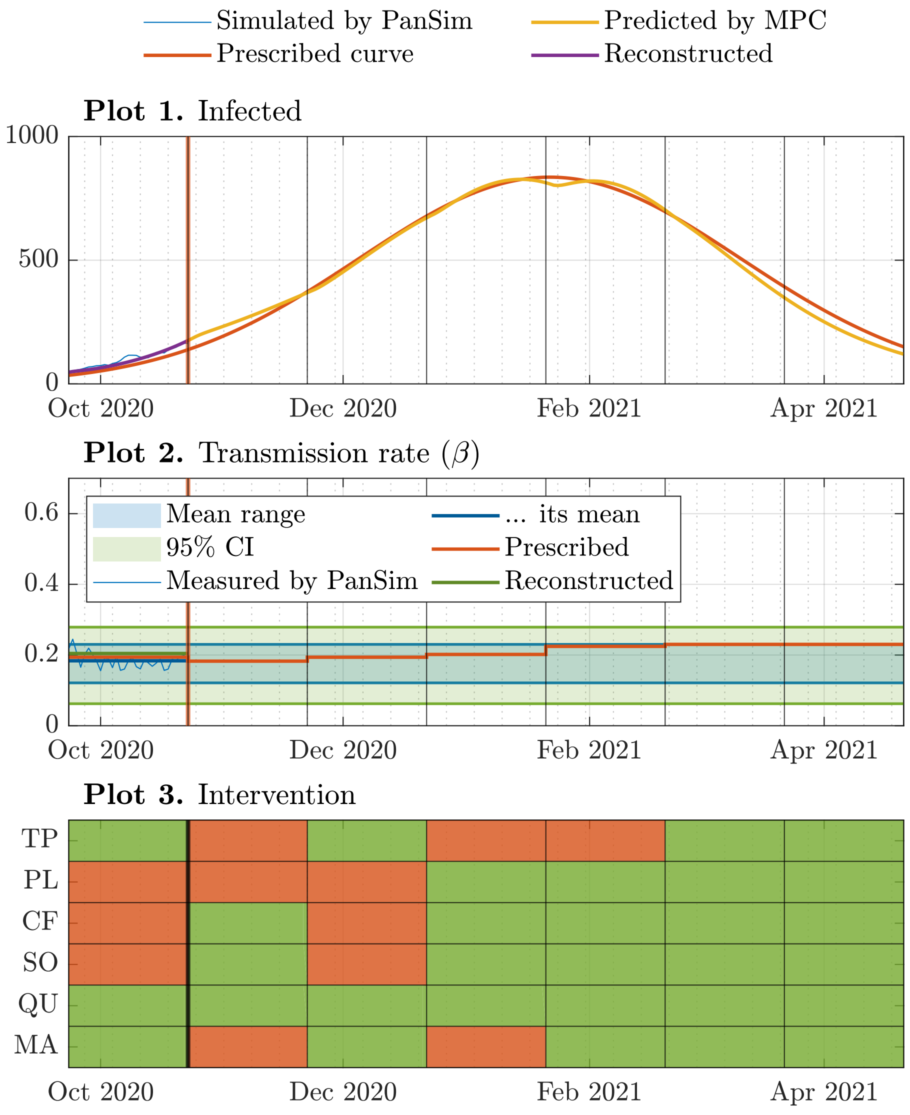


**Figure 6. Control simulation before the second intervention planing cycle.** The vertical red line illustrates the current time of the simulation, which is now at **day** 30. The red bell-shaped curve illustrates the prescribed trajectory for the infected people with symptoms in the main phase of the disease (**I**). The light blue signals in Plot 1 and Plot 2 illustrate the simulated value of **I** and the numerically approximated transmission rate of the simulated spread. Purple signal in Plot 1 constitutes the reconstructed number of infected **I** if a constant (but potentially different) transmission rate is assumed for each cycles (solid green staircase function in Plot 2). In Plot 2. the red staircase function illustrates the necessary transmission rate $\beta$ to achieve the prescribed curve for **I**. The blue area in Plot 2 highlights the range of the computed mean values for $\beta$ for the different interventions, whereas, the green area illustrates the range of the mean $\pm$ Std computed for the different interventions. Plot 3 illustrates the stringency (low: green, medium: yellow, high: red) planned for the six independent measures summarized in Table 1 of the manuscript. The reference trajectory (red bell-shaped curve) is the same as in Figure 5, but the prediction made from day 30 for the remaining $T = 6 \times h = 180\,[\text{days}]$ is slightly different from that in Figure 5. This is due to the fact that in the first $h = 30\,[\text{days}]$ the simulated epidemic process did not follow the predicted curve exactly. Accordingly, the transmission rates and the interventions, respectively, are recalculated from day 31.

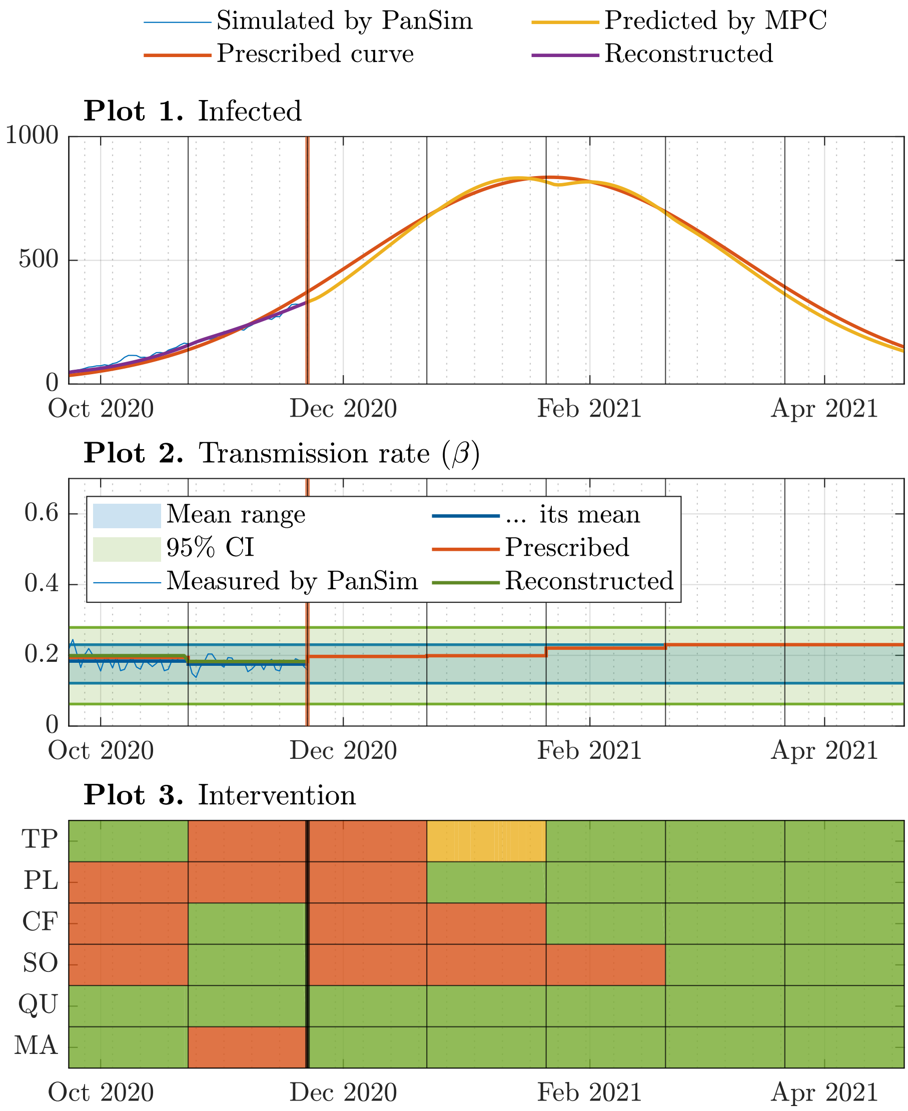


**Figure 7. Control simulation before the third intervention planing cycle.** The vertical red line illustrates the current time of the simulation, which is now at day $2h = 60$ [days]. The light blue signals in Plot 1 and Plot 2 illustrate the simulated value of $\mathbf{I}$ and the numerically approximated transmission rate of the simulated spread. Purple signal in Plot 1 constitutes the reconstructed number of infected $\mathbf{I}$ if a constant (but potentially different) transmission rate is assumed for each cycles (solid green staircase function in Plot 2). The reference trajectory (red bell-shaped curve) is the same as in Figure 5, but the prediction made from day 60 for the remaining $T = 5 \times h = 150$ [days] is slightly different from that in Figure 6. This is due to the fact that in the second cycle the simulated epidemic process did not follow the curve predicted at day 30 exactly. Accordingly, the transmission rates and the interventions, respectively, are recalculated from day $2h + 1 = 61$.

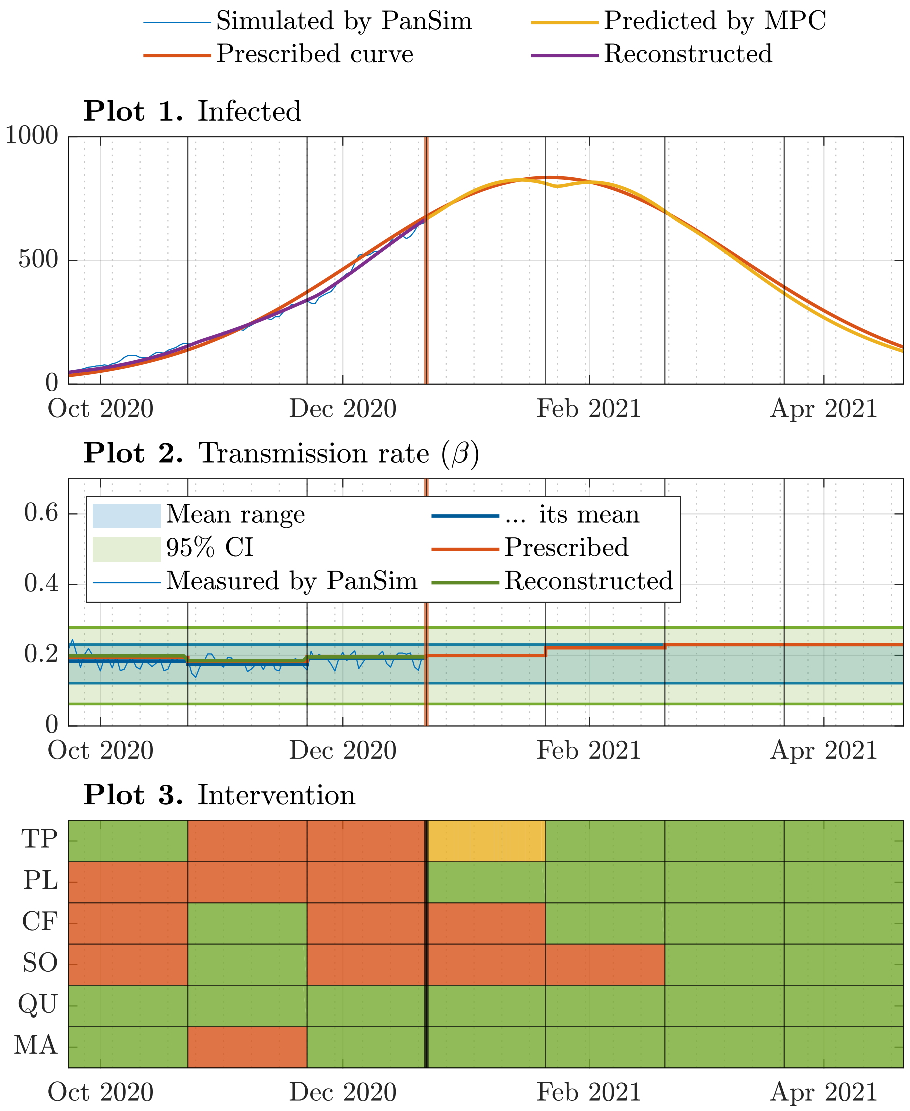


**Figure 8. Control simulation before the 4th intervention planing cycle.** The vertical red line illustrates the current time of the simulation. We refer to Figures 5, 6, 7 for more details.

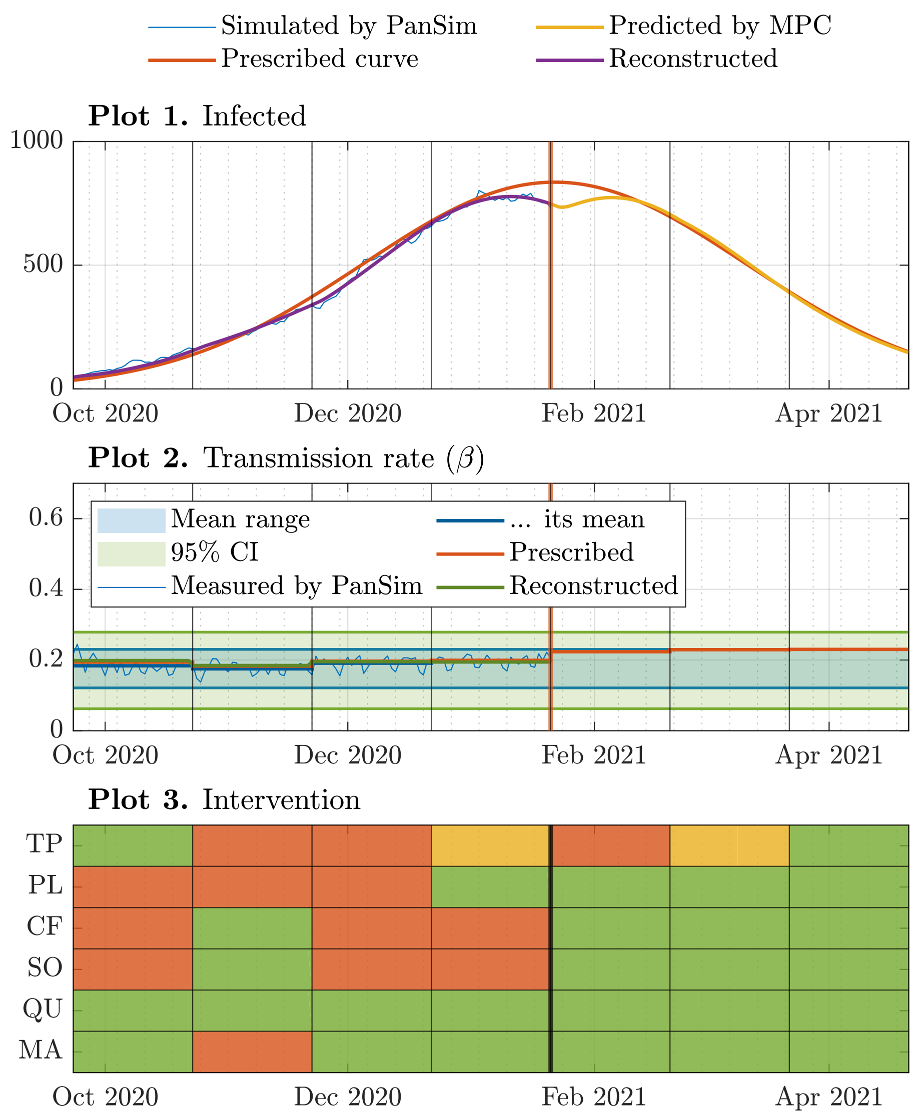


**Figure 9. Control simulation before the 5th intervention planing cycle.** The vertical red line illustrates the current time of the simulation. We refer to Figures 5–8 for more details.

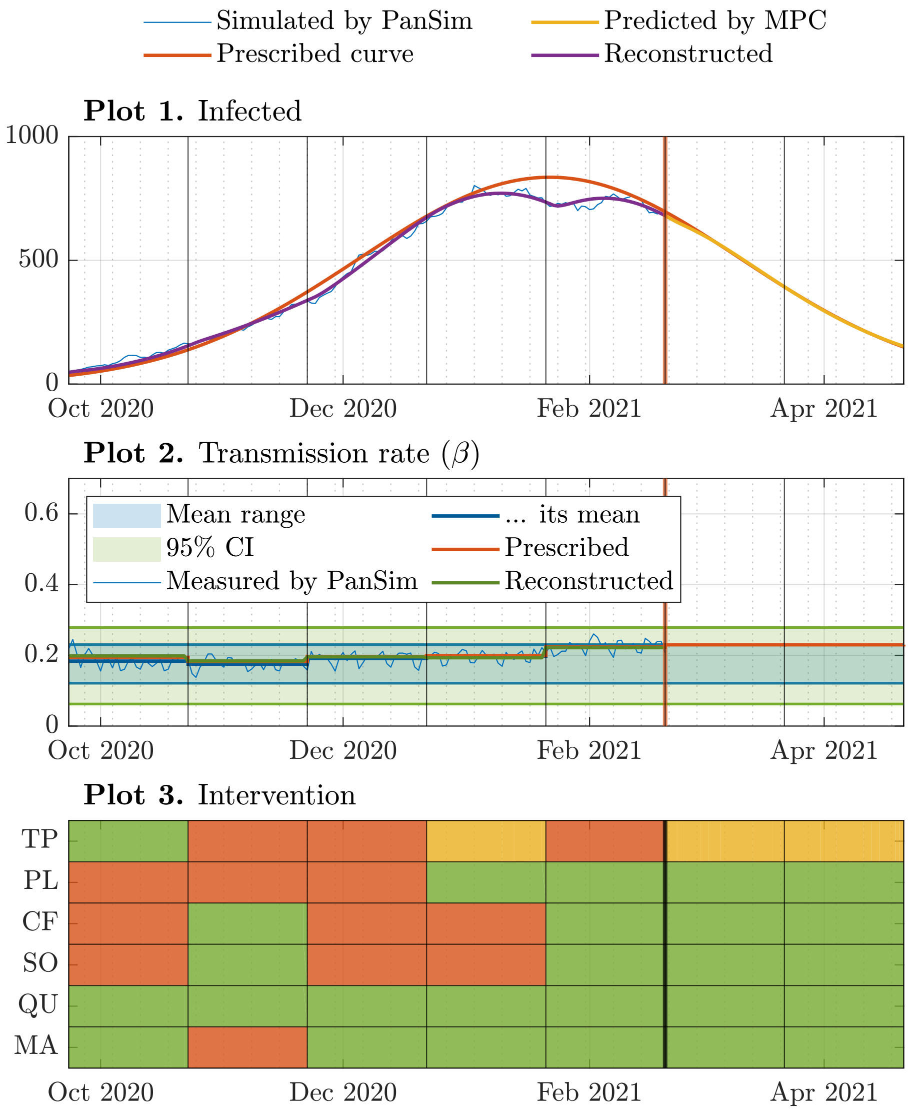


**Figure 10. Control simulation before the 6th intervention planing cycle.** The vertical red line illustrates the current time of the simulation. We refer to Figures 5–9 for more details.

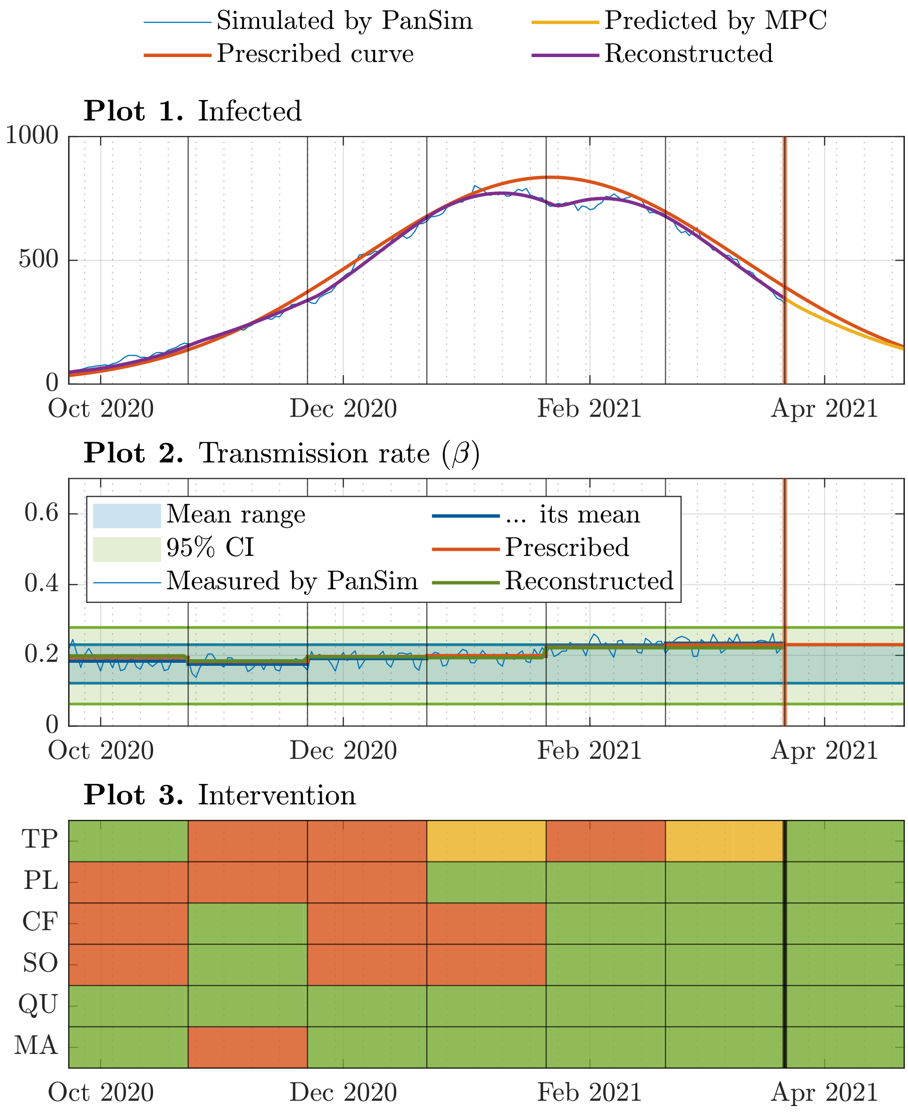


**Figure 11. Control simulation before the 7th intervention planing cycle.** The vertical red line illustrates the current time of the simulation. We refer to Figures 5–10 for more details.

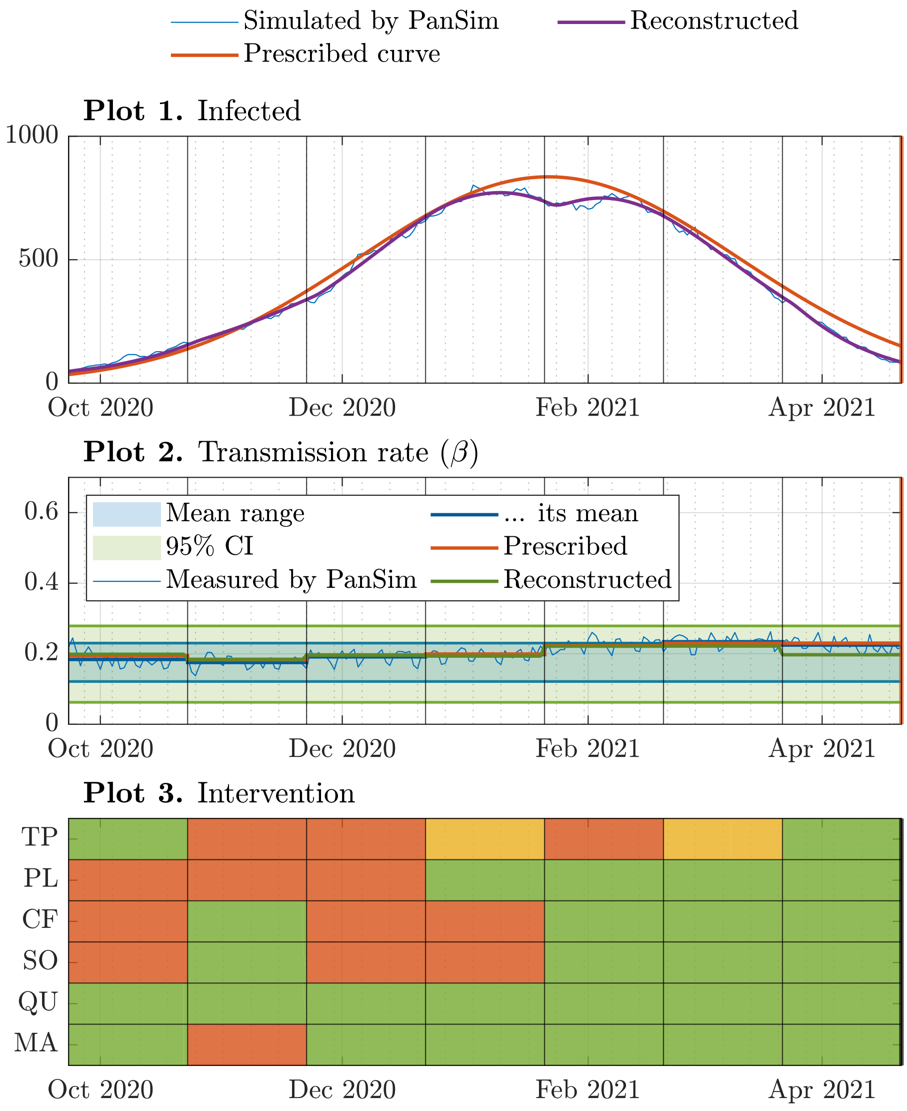


**Figure 12. Control simulation after the 7th intervention planing cycle.** The vertical red line illustrates the current time of the simulation. We refer to Figures 5–11 for more details.

# References


[1] T. Hale et al. "A global panel database of pandemic policies (Oxford COVID-19 government response tracker)". In: *Nature Human Behaviour* 5.4 (Mar. 2021), pp. 529–538. DOI: 10.1038/s41562-021-01079-8.

[2] J.-H. Niemann et al. "Koopman-based surrogate models for multi-objective optimization of agent-based systems". In: *Physica D: Nonlinear Phenomena* 460 (Apr. 2024), p. 134052. ISSN: 0167-2789. DOI: https://doi.org/10.1016/j.physd.2024.134052.

[3] H. Ryu, A. Abulali, and S. Lee. "Assessing the effectiveness of isolation and contact-tracing interventions for early transmission dynamics of COVID-19 in South Korea". In: *IEEE Access* 9 (2021), pp. 41456–41467. DOI: 10.1109/ACCESS.2021.3064371.

[4] P. Polcz, B. Csutak, and G. Szederkényi. "Reconstruction of epidemiological data in Hungary using stochastic model predictive control". In: *Applied Sciences* 12.3 (2022), p. 1113. ISSN: 2076-3417. DOI: 10.3390/app12031113.

[5] G. An et al. "Optimization and control of agent-based models in biology: A perspective". In: *Bulletin of Mathematical Biology* 79.1 (Nov. 2016), pp. 63–87. ISSN: 1522-9602. DOI: 10.1007/s11538-016-0225-6.

[6] T. Péni et al. "Nonlinear model predictive control with logic constraints for COVID-19 management". In: *Nonlinear Dynamics* 102.4 (Dec. 2020), pp. 1965–1986. DOI: 10.1007/s11071-020-05980-1.

[7] I. Z. Reguly et al. "Microsimulation based quantitative analysis of COVID-19 management strategies". In: *PLOS Computational Biology* 18.1 (Jan. 2022), pp. 1–14. DOI: 10.1371/journal.pcbi.1009693.

[8] B. Keomley-Horvath et al. "The design and utilisation of PanSim, a portable pandemic simulator". In: *2022 First Combined International Workshop on Interactive Urgent Supercomputing (CIW-IUS)*. IEEE, Nov. 2022. DOI: 10.1109/ciw-ius56691.2022.00006.

[9] P. Polcz et al. "Wastewater-based modeling, reconstruction, and prediction for COVID-19 outbreaks in Hungary caused by highly immune evasive variants". In: *Water Research* 241 (May 2023), p. 120098. ISSN: 0043-1354. DOI: 10.1016/j.watres.2023.120098.

[10] A. C. S. Jørgensen et al. "Efficient Bayesian inference for stochastic agent-based models". In: *PLOS Computational Biology* 18.10 (Oct. 2022). Ed. by M. Alber, e1009508. ISSN: 1553-7358. DOI: https://doi.org/10.1371/journal.pcbi.1009508.

[11] C. Angione, E. Silverman, and E. Yaneske. "Using machine learning as a surrogate model for agent-based simulations". In: *PLOS ONE* 17.2 (Feb. 2022). Ed. by R. Bouffanais, e0263150. ISSN: 1932-6203. DOI: 10.1371/journal.pone.0263150.

[12] J.-H. Niemann et al. "Multilevel optimization for policy design with agent-based epidemic models". In: *Journal of Computational Science* 77 (Apr. 2024), p. 102242. ISSN: 1877-7503. DOI: 10.1016/j.jocs.2024.102242.

[13] L. L. Fonseca et al. "Optimal control of agent-based models via surrogate modeling". In: *PLOS Computational Biology* 21.1 (Jan. 2025). Ed. by M. Alber, e1012138. ISSN: 1553-7358. DOI: 10.1371/journal.pcbi.1012138.
